# Ral-Arf6 crosstalk regulates Ral dependent exocyst trafficking and anchorage independent growth signalling

**DOI:** 10.1016/j.cellsig.2016.05.023

**Published:** 2016-09

**Authors:** Archana Pawar, Jeremy A. Meier, Anwesha Dasgupta, Neha Diwanji, Neha Deshpande, Kritika Saxena, Natasha Buwa, Siddhi Inchanalkar, Martin Alexander Schwartz, Nagaraj Balasubramanian

**Affiliations:** a Indian Institute of Science Education and Research, Dr. Homi Bhabha Road, Pashan, Pune 411 008, Maharashtra, India; b Robert M. Berne Cardiovascular Research Center, University of Virginia, Charlottesville, VA 22908, United States; c Yale Cardiovascular Research Center, 300 George Street, 7th Floor, New Haven, CT 06511, United States

**Keywords:** Adhesion, Ral, Arf6, Exocyst trafficking, Anchorage independence, Cancer

## Abstract

Integrin dependent regulation of growth factor signalling confers anchorage dependence that is deregulated in cancers. Downstream of integrins and oncogenic Ras the small GTPase Ral is a vital mediator of adhesion dependent trafficking and signalling. This study identifies a novel regulatory crosstalk between Ral and Arf6 that controls Ral function in cells. In re-adherent mouse fibroblasts (MEFs) integrin dependent activation of RalA drives Arf6 activation. Independent of adhesion constitutively active RalA and RalB could both however activate Arf6. This is further conserved in oncogenic H-Ras containing bladder cancer T24 cells, which express anchorage independent active Ral that supports Arf6 activation. Arf6 mediates active Ral-exocyst dependent delivery of raft microdomains to the plasma membrane that supports anchorage independent growth signalling. Accordingly in T24 cells the RalB-Arf6 crosstalk is seen to preferentially regulate anchorage independent Erk signalling. Active Ral we further find uses a Ral-RalBP1-ARNO-Arf6 pathway to mediate Arf6 activation. This study hence identifies Arf6, through this regulatory crosstalk, to be a key downstream mediator of Ral isoform function along adhesion dependent pathways in normal and cancer cells.

## Highlights

• Ral mediates Arf6 activation downstream of integrins and oncogenic Ras.
• Arf6 mediates Ral-exocyst dependent delivery of raft microdomains.
• Active Ral supports anchorage independent Arf6 activation in bladder cancer T24 cells.
• Ral-Arf6 crosstalk in T24 cells regulates anchorage independent Erk signalling.
• A Ral-RalBP1-ARNO-Arf6 pathway mediates the Ral-Arf6 crosstalk.

## 1 Introduction

Anchorage independent signalling in cancer cells is a key component of cancer cell invasion and metastasis, reflecting the ability of cancer cells to survive and grow in diverse and otherwise unfavourable environments. Cancer metastasis is a major cause of mortality, and thus understanding the fundamental basis for anchorage dependence and how it is overcome in cancers is an important problem. In Ras-dependent cancers, anchorage independence is induced largely through the Ras-RalGEF-Ral pathway, in which the Ras related GTPases RalA and RalB are the key mediators [1], [2]. Ral GTPases are also activated in Ras-independent cancers through other mechanisms [3], [4], [5].Ral isoforms, RalA and RalB, though 82% identical regulate distinct cellular functions in normal and cancer cells [6], [7], [8], [9]. RalA is implicated in driving anchorage independence [5], [10]while RalB supports cell survival in cancers [11]. RalA and RalB bind the same effectors but can signal differently, mediated by their differential activation [6], utilization of downstream effectors [1]and/or localization in cells [8], [12], [13].

Anchorage dependence in cells is mediated by integrin-dependent regulation of cell cycle progression. One key aspect of this regulation is the ability of integrin mediated adhesion to control growth factor receptor signalling [14], [15]. The trafficking and cell membrane targeting of raft microdomains that act as anchoring sites for signalling molecules on the plasma membrane is a major mechanism for this regulation [16], [17]. Detaching cells from the extracellular matrix (ECM) triggers rapid endocytosis of these microdomains through caveolae [17], which dramatically decreases plasma membrane order [18] and inhibits signalling by Erk, PI3-kinase and Rho GTPases [16], [17], [19]. Replating of cells on ECM triggers rapid activation of Arf6 and RalA, which drive the return of these microdomains to the plasma membrane to restore anchorage dependent signalling [19], [20]. Exocytosis of these vesicles is mediated by the exocyst complex, which both Ral [21], [22], [23]and Arf6 [24]bind to. In mouse fibroblasts, active RalA is necessary and sufficient to drive this pathway whereas Arf6 is necessary but not sufficient [19], [20]. Interestingly, Ral and Arf6 also jointly regulate phospholipase D (PLD) activation [25], [26],GLUT4 receptor trafficking [27], [28], [29], [30], insulin secretion [27], [31], [32] and Fc-gamma-R mediated phagocytosis [33], [34].

These considerations led us to investigate the relationship between the small GTPases Ral and Arf6 in adhesion-dependent signalling in normal cells and anchorage-independent signalling in Ras transformed cancer cells. This study identifies a regulatory crosstalk between Ral and Arf6 that drives their activation downstream of both integrins and oncogenic Ras. Both active RalA and RalB can support Arf6 activation, with their variable activation by different stimuli differentially regulating this crosstalk and downstream signalling.

## 2 Results

### 2.1 RalA regulates adhesion dependent activation of Arf6 in MEFs

**Fig. 1:**
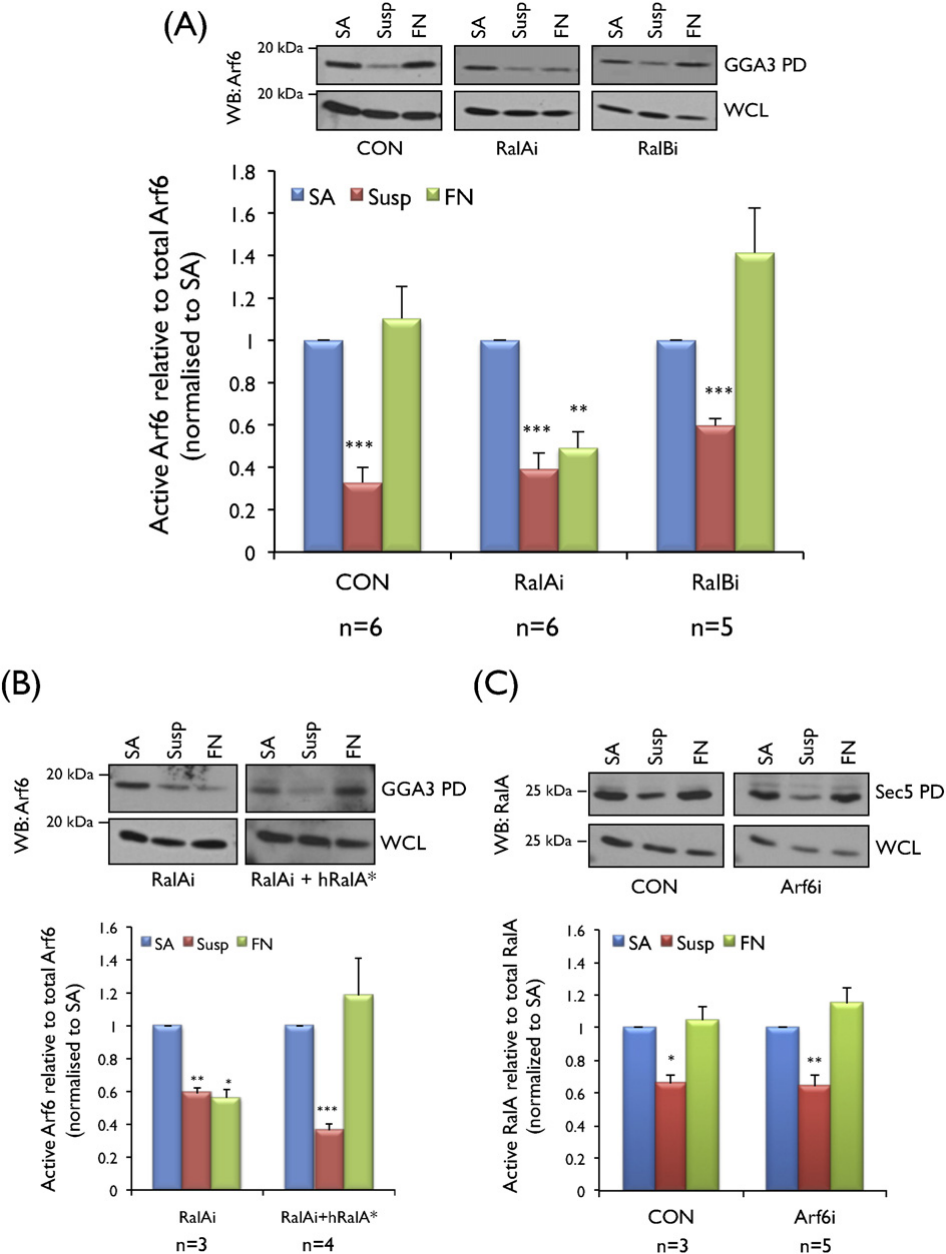
**RalA**, **but not RalB regulates adhesion dependent activation of Arf6**. Western blot detection and quantitation of active Arf6 pulled down by GST-GGA3 (GGA3 PD) and total Arf6 in whole cell lysate (WCL) was done from low serum (**A**) control (CON), RalA knockdown (RalAi) and RalB knockdown (RalBi) MEFs which were stable adherent (SA), suspended (Susp) or readherent on fibronectin (FN). (**B**) Arf6 activity was similarly detected and compared from low serum RalA knockdown MEFs (RalAi) reconstituted with siRNA resistant HA-hRalA* (RalAi + hRalA*). (**C**) Similar western blot detection and quantitation of active RalA pulled down by GST-Sec5 (Sec5 PD) and total RalA in whole cell lysate (WCL) from low serum control (CON) and Arf6 knockdown MEFs (Arf6i). Calculated percentage active Arf6 and RalA levels were normalized to respective SA. Graph represents mean ± standard error from a minimum of three and maximum of six independent experiments (as indicated below each graph). Statistical analysis of all the data was done using the two tailed single sample *t*-test and their significance represented (* *p* value < 0.05, ** *p* value < 0.01 and *** *p* value < 0.001).

Our previous studies showed that RalA and Arf6 (but not RalB) activity decreases on loss of adhesion and recovers upon re-adhesion to fibronectin [19], [20]. Active RalA and Arf6 promoted exocyst-dependent trafficking and plasma membrane delivery of raft microdomains to support anchorage dependent signalling [20]. Active Arf6 while necessary is not sufficient for this exocytic delivery while active RalA is necessary and sufficient [19], [20]. To evaluate the possible association between these GTPases, we measured the activation of each GTPase during integrin-mediated adhesion on siRNA mediated knockdown of the other. RalA knockdown (Fig. S1A) did not affect the drop in Arf6 activity on loss of adhesion but prevented its recovery on re-adhesion to fibronectin (Fig. 1A). Reconstitution of RalA knockdown cells with a siRNA resistant mutant of human RalA [20] (Fig. S1D) restored normal Arf6 activation (Fig. 1B). By contrast, siRNA mediated RalB knockdown (Fig. S1B) did not affect Arf6 activation (Fig. 1A). Arf6 knockdown (Fig. S1C) also had no effect on RalA activation (Fig. 1C) suggesting the presence of an integrin-RalA-Arf6 pathway in MEFs.

### 2.2 Active Ral supports anchorage independent Arf6 activation and membrane exocytosis

**Fig. 2:**
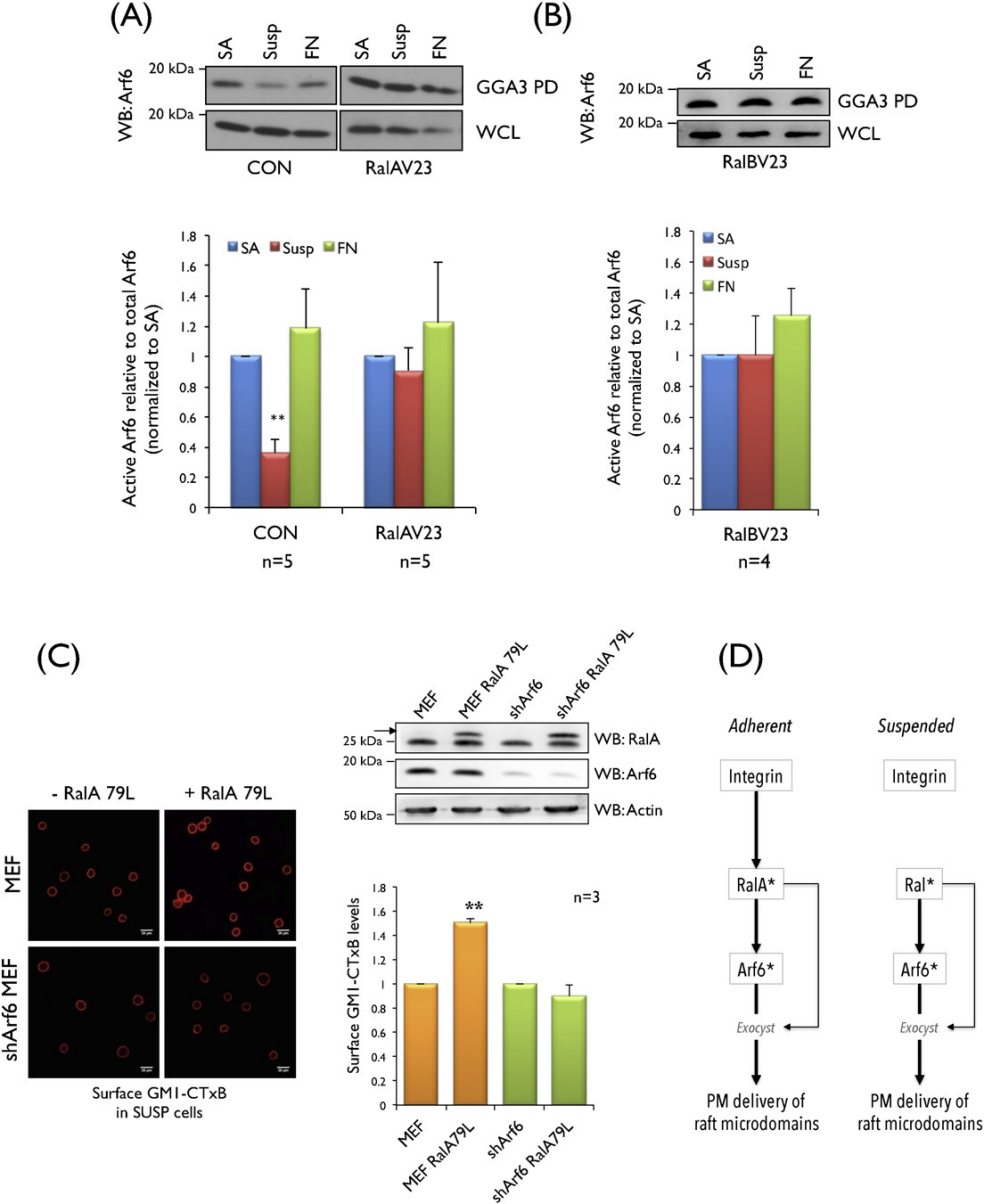
**Constitutively active Ral supports anchorage independent Arf6 activation needed for membrane raft exocytosis**. Western blot detection and quantitation of active Arf6 pulled down by GST-GGA3 (GGA3 PD) and total Arf6 in whole cell lysate (WCL) was done from low serum control (CON) and (**A**) active RalA (RalAV23) and (**B**) active RalB (RalBV23) expressing MEFs which were stable adherent (SA), suspended (Susp) or readherent on fibronectin (FN). Calculated percentage active Arf6 levels were normalized to respective SA. Graph represents mean ± standard error from a minimum of four and maximum of five independent experiments (as indicated below each graph). (**C**) Expression of the FLAG tagged RalA mutant (R79L) (WB: RalA) (marked by arrow) in control (MEF) and stable shRNA Arf6 expressing MEFs (shArf6). Loss of Arf6 (WB: Arf6) relative to actin (WB: Actin) was confirmed. Cell surface GM1 bound CTxB-Alexa 594 was imaged (left panel) and intensity quantitated by measuring integrated density. Mean integrated density for a minimum of 50 cells was calculated and normalized to their respective controls. Graph is mean ± standard error from 3 such independent experiments. (**A**, **B**, **C**) Statistical analysis of normalized data was done using the two tailed single sample *t*-test and their significance represented (* *p* value < 0.05 and ** *p* value < 0.01). (**D**) Schematic represents the linear integrin-RalA-Arf6 pathway (bold arrows) along with the contribution active Ral (RalA*) makes with active Arf6 (Arf6*) (lighter arrow) in mediating the plasma membrane (PM) delivery of raft microdomains through the exocyst complex.

We next investigated this pathway in anchorage independent conditions to further evaluate the contribution integrins make. Active RalA in these conditions drives membrane raft exocytosis and anchorage independent signalling in mouse fibroblasts [20]. Expression of constitutively active RalA (RalAV23) in MEFs (Fig. S1E, S1G) induced anchorage independent Arf6 activation (Fig. 2A) indicating that RalA can mimic the effect of integrins along this pathway. Interestingly, constitutively active RalB (RalBV23) also induced anchorage independent Arf6 activation (Fig. 2B, S1F, S1H). This suggests the presence of a Ral-Arf6 crosstalk in cells, with a major role for RalA downstream of integrins, a possible consequence of its differential activation in re-adherent MEFs. It also establishes active GTP bound Ral as the mediator of Arf6 activation, further suggesting that this crosstalk could exist downstream of other stimuli that can activate Ral. We next evaluated the functional significance of the Ral-Arf6 crosstalk, by testing its role in the exocyst dependent membrane raft trafficking in non-adherent MEFs [20]. Expression of the fast cycling RalA (79L) mutant in these cells is seen to promote anchorage-independent plasma membrane delivery of GM1 containing raft microdomains [20]. Stable knockdown of Arf6 (Fig. S1I) while only modestly affecting total surface GM1 levels (Fig. S1J) did block the ability of active RalA (79L) to deliver GM1 containing raft microdomains to the plasma membrane in non-adherent MEFs (Fig. 2C). This establishes a functional role for Arf6 activated downstream of Ral and hence for this crosstalk in RalA-exocyst mediated membrane raft trafficking. Our earlier studies have shown active Arf6 by itself could not mediate the complete delivery of raft microdomains at the plasma membrane [19]. This suggests that along with the linear integrin-Ral-Arf6 pathway (bolder arrows), RalA works with active Arf6 to deliver raft microdomains to the plasma membrane (thin arrow), possibly through the exocyst complex (Fig. 2D). In vitro studies have shown GST-RalA can bind Arf proteins [35] and they colocalize in lighter membrane raft fractions in H-Ras transformed cells [25], [35]. When expressed in HEK293T cells WT and active RalA and RalB both comparably bound active T157A Arf6 (Fig. S1K), suggesting their association to not be a direct effector interaction, but possibly mediated by a Ral effector. When expressed at levels comparable or less than endogenous protein (Fig. S1M), wild type and active RalA/RalB (V23) also colocalize with active Arf6 (T157A) in membrane ruffles of re-adherent MEFs (Fig. S1L) further supporting the plasma membrane to be an active site for this crosstalk in cells.

### 2.3 Ral –Arf6 crosstalk in Ras transformed cancers

**Fig. 3:**
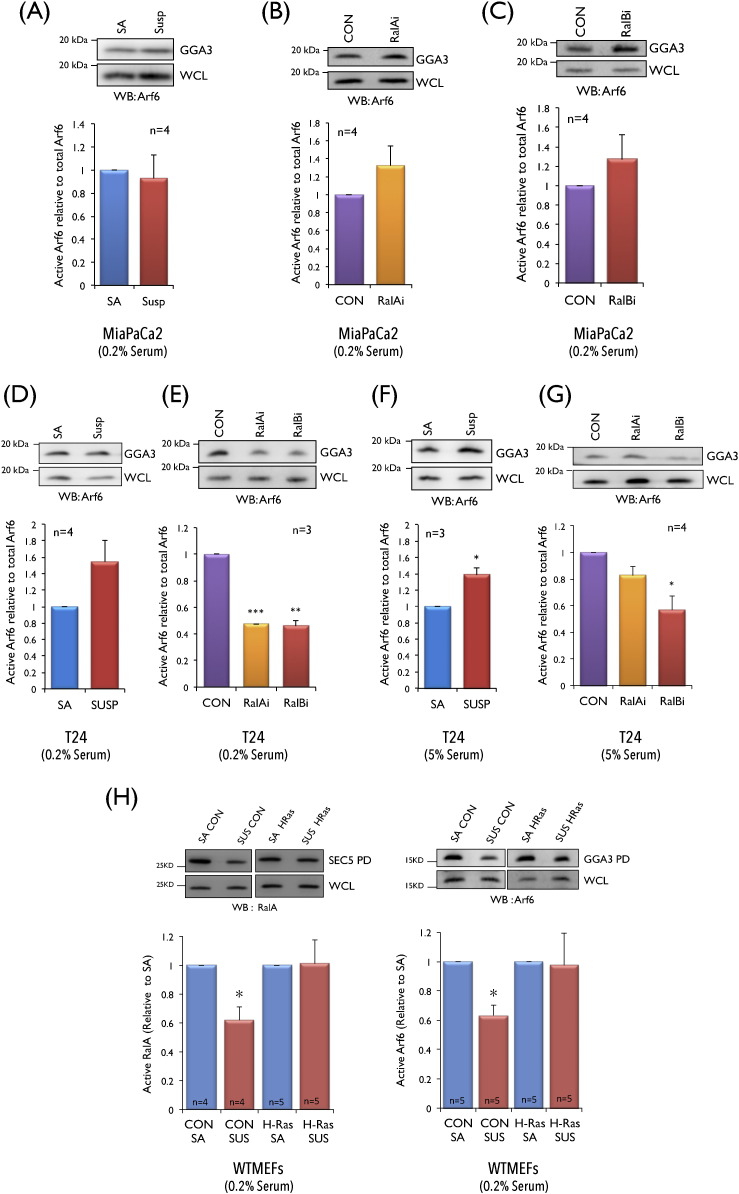
**Ral**-**Arf6 crosstalk in Ras**-**transformed MiaPaCa2 and T24 cells**. (**A**–**C**) Western blot detection and quantitation of active Arf6 pulled down by GST-GGA3 (GGA3) and total Arf6 in the respective whole cell lysate (WCL) from MiaPaCa2 cells grown with low serum(**A**) stable adherent (SA) and suspended (Susp), (**B**) suspended control (CON) and RalA knockdown (RalAi) and (**C**) suspended control (CON) and RalB knockdown (RalBi). (**D**–**G**) Western blot detection and quantitation of active Arf6 from T24 cells stable adherent (SA) and suspended for 120 min (Susp) (**D**) with low serum and (**F**) with serum. Similar Active Arf6 levels were determined in control (CON), RalA knockdown (RalAi) and RalB knockdown (RalBi) T24 cells suspended for 120 min (**E**) with low serum and (**G**) with serum. (**H**) Western blot detection and quantitation of active Arf6 and active RalA from WTMEFs stable adherent (SA) and suspended for 120 min (SUS) in the absence (CON) and presence of G12V H-Ras (H-Ras) with low serum. Calculated percentage active Arf6 levels and RalA levels were normalized to respective SA/CON. Graphs represent mean ± standard error from a minimum of three and maximum of five independent experiments as indicated in each graph. Statistical analysis of normalized data was done using the two tailed single sample *t*-test and their significance represented (* *p* value < 0.05, ** *p* value < 0.01 and *** *p* value < 0.001).

The Ras-RalGEF-Ral pathway [5], [36], [37] supports anchorage independent Ral activation in cancer [10]. In oncogenic Ras driven cancers, RalA is the principal determinant of anchorage independent signalling and growth [5], [36], [37]whereas RalB is critical for cell survival [11], [38], [39]. RalB can however also contribute to anchorage independence in some cancers [40]. We therefore examined the role of RalA and RalB in regulating Arf6 in K-Ras expressing pancreatic (MiaPaCa2) and H-Ras expressing bladder (T24) cancer cells [37], [41]. We observed anchorage independent activation of RalA, RalB (Fig. S2A, S2B, S2C, S2G) and Arf6, (Fig. 3A and D, S2D) in both cell lines. However, siRNA mediated knockdown of RalA and RalB (Fig. S2E, S2F) in MiaPaCa2 cells did not affect anchorage independent Arf6 activation (Fig. 3B and C). In T24 cells loss of RalA and RalB (Fig. S2H) did significantly and comparably reduce (by > 50%) anchorage independent Arf6 activity (Fig. 3E). Thus, while both Ral and Arf6 are anchorage-independent in these cancer cells their regulatory crosstalk is clearly different. In T24 cells, RalA and RalB act upstream of Arf6 but not in MiaPaCa2 cells.

To further evaluate ability of oncogenic H-Ras to support the Ral-Arf6 pathway in these cells, we tested and found the expression of G12V H-Ras in anchorage dependent WTMEFs (Fig. S2J) to promote anchorage independent activation of RalA and Arf6 (Fig. 3H). Considering expression of active RalAV23 is seen to similarly drive anchorage independent Arf6 activation in WTMEFs (Fig. 2A), these observations suggest the presence of a H-Ras-Ral-Arf6 pathway.

The above studies looking at the Ral-Arf6 crosstalk in T24 cells (Fig. 3D, E) were done in serum starved (low serum) conditions so that integrin-mediated adhesion could be observed as the primary mediator of signalling. Knowing the existence of integrin-growth factor synergies [14] we further tested this in the presence of serum, aware that anchorage independent growth studies implicating Ral in cancers were also done with serum [37], [42]. Detachment of T24 cells in the presence of serum also showed increased Arf6 activation relative to stable adherent cells (Fig. 3F). This anchorage-independent Arf6 activity was reduced significantly by loss of RalB but not RalA (Fig. 3G, S2I). This difference in regulation between RalA/RalB is not seen in T24 cells with low serum (Fig. 3E). These results hence support the general conservation of a H-Ras-Ral-Arf6 pathway in bladder cancer cells but illustrate its differential regulation by integrins and growth factors. It also suggests that Ral isoforms in this background can differentially regulate this crosstalk that could possibly contribute to their differential function in these cells.

### 2.4 Role of Ral-Arf6 crosstalk in anchorage independent signalling in cancers

**Fig. 4:**
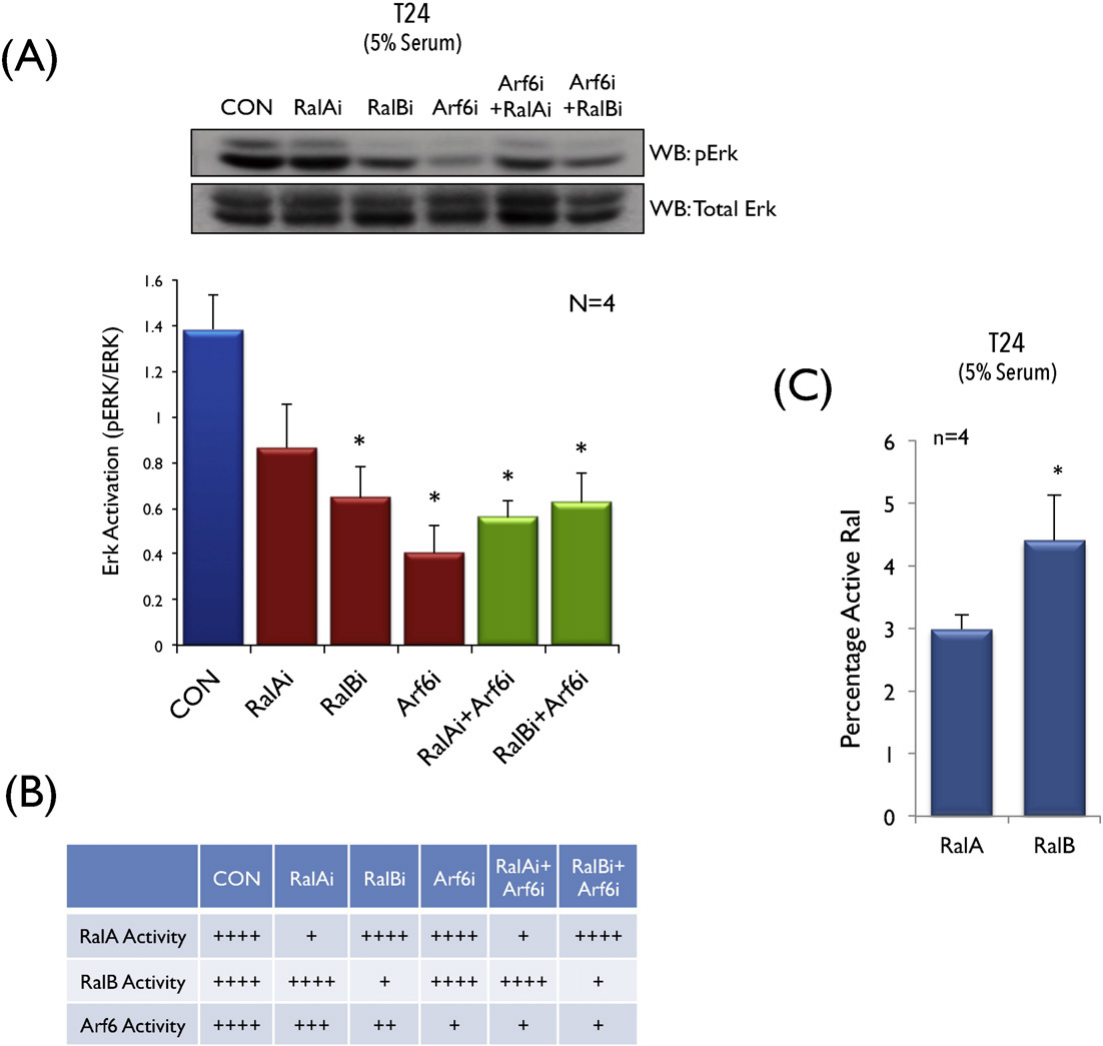
**Ral**-**Arf6 crosstalk regulates anchorage independent Erk signalling in T24 cells** (**A**) Western blot detection and quantitation of Erk1/2 phosphorylation (P-p44/42 MAPK-Thr202/Tyr204) (WB:pErk) relative to total Erk1/2 (p44/42-MAPK) (WB:Erk) in whole cell lysates from control (CON), RalA knockdown (RalAi), RalB knockdown (RalBi), Arf6 knockdown (Arf6i), combined RalA and Arf6 knockdown (RalAi + Arf6i) and combined RalB and Arf6 knockdown (RalBi + Arf6i) T24 cells held in suspension with serum for 120 min. Graph represents mean ± standard error of the pErk/total Erk band intensity ratios from four independent experiments. Statistical analysis was done comparing knockdown samples to the control (CON) using the unpaired Students's *t*-test (* *p*-value < 0.05). (**B**) Table consolidates changes in activity of RalA, RalB and Arf6 based on the effect of their respective knockdowns and crosstalk in the presence of serum (Fig. 4G). Basal activation is represented as ++++ and relative changes indicated accordingly. (**C**) Graph represents percentage active RalA and RalB levels in T24 cells suspended with serum from 4 independent experiments (mean ± standard error). This data is analyzed using the unpaired Student's *t*-test (* *p*-value < 0.05).

We hence further explored the role of the RalA/RalB-Arf6 crosstalk in anchorage independent Erk signalling (Fig. 4A). Active Ral-exocyst mediated membrane raft trafficking in our earlier studies is seen to regulate anchorage independent Erk activation [20]. To test this regulation in T24 cells we did both single and joint knockdowns of Ral and Arf6 in the presence of serum (confirmed in Fig. S3A, S3B, S3C). Erk activation (Phospho-p44/42 MAPK (Thr202/Tyr204)) in non-adherent T24 cells was significantly reduced by the depletion of Arf6, reflecting its role at the end of the linear H-Ras-Ral-Arf6 pathway. We find Erk activation to be only modestly affected by loss of RalA (Fig. 4A), reflecting the marginal effect its knockdown has on Arf6 activation in non-adherent T24 cells with serum (Fig. 3G). Their combined knockdown reduced Erk activation significantly (Fig. 4A) further reflecting the role Arf6 has downstream of Ral. Interestingly, loss of RalB seen to significantly reduce Arf6 activation in suspended T24 cells with serum (Fig. 3G) did significantly affect anchorage independent Erk activation, comparable to its joint knockdown with Arf6 (RalBi + Arf6i)(Fig. 4A and B, S3). This explains the differential effect RalB and RalA have on Erk activation in T24 cells, which is lost on their joint knockdown with Arf6. This could further be mediated by the differential activation RalB in these cells. In bladder cancers RalB is reported to be more active than RalA in some studies [41], [43], with a reported role in migration, proliferation and anchorage independence as well [40], [42], [44]. In non-adherent T24 cells we tested and found RalB activity was indeed significantly higher than RalA (Fig. 4C). This could mediate its differential utilization of Ral effectors to differentially regulate Arf6 activation (Fig. 3G) and anchorage independent Erk signalling (Fig. 4A).

### 2.5 Role of Ral effectors in mediating Ral-Arf6 crosstalk

**Fig. 5:**
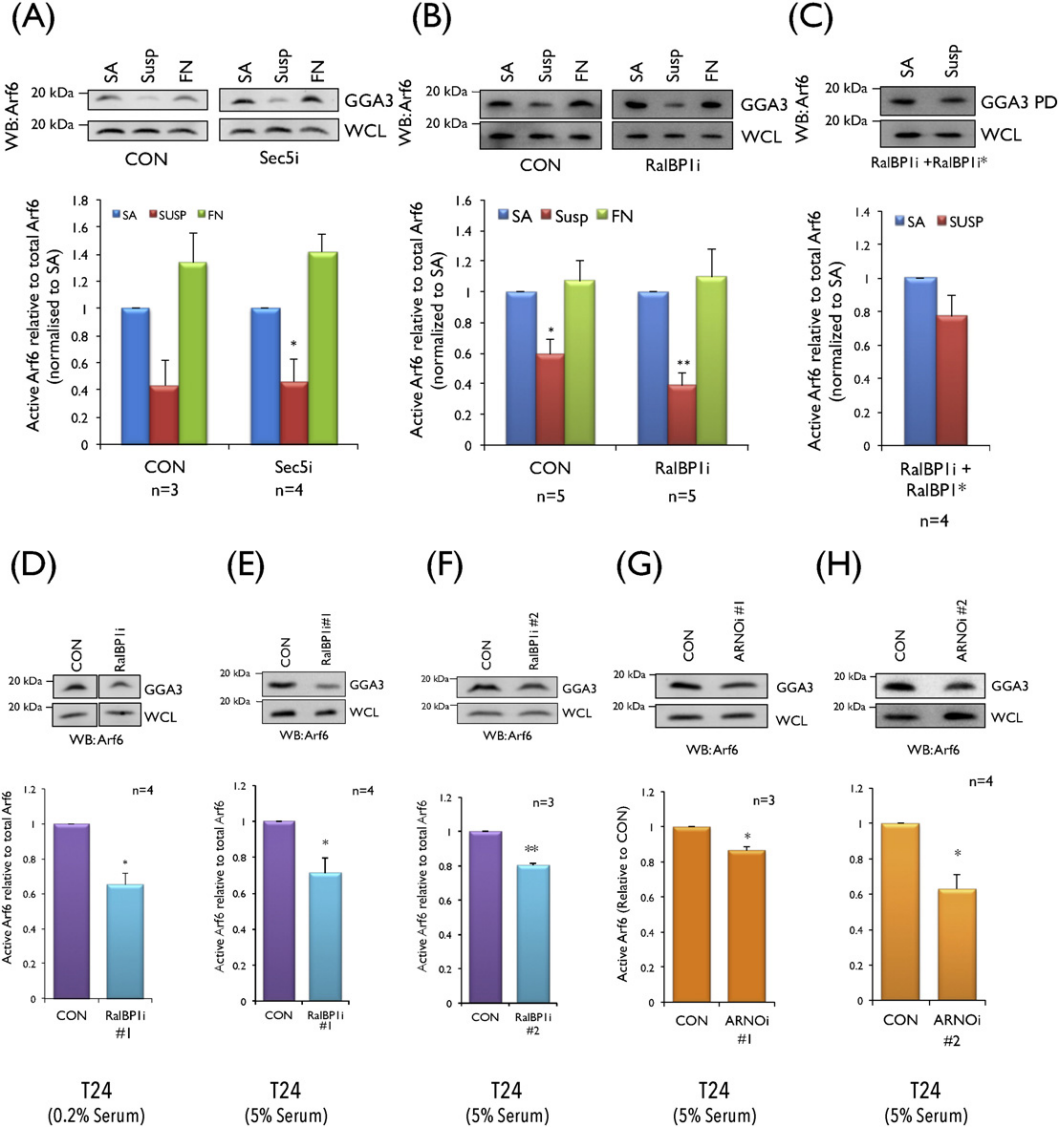

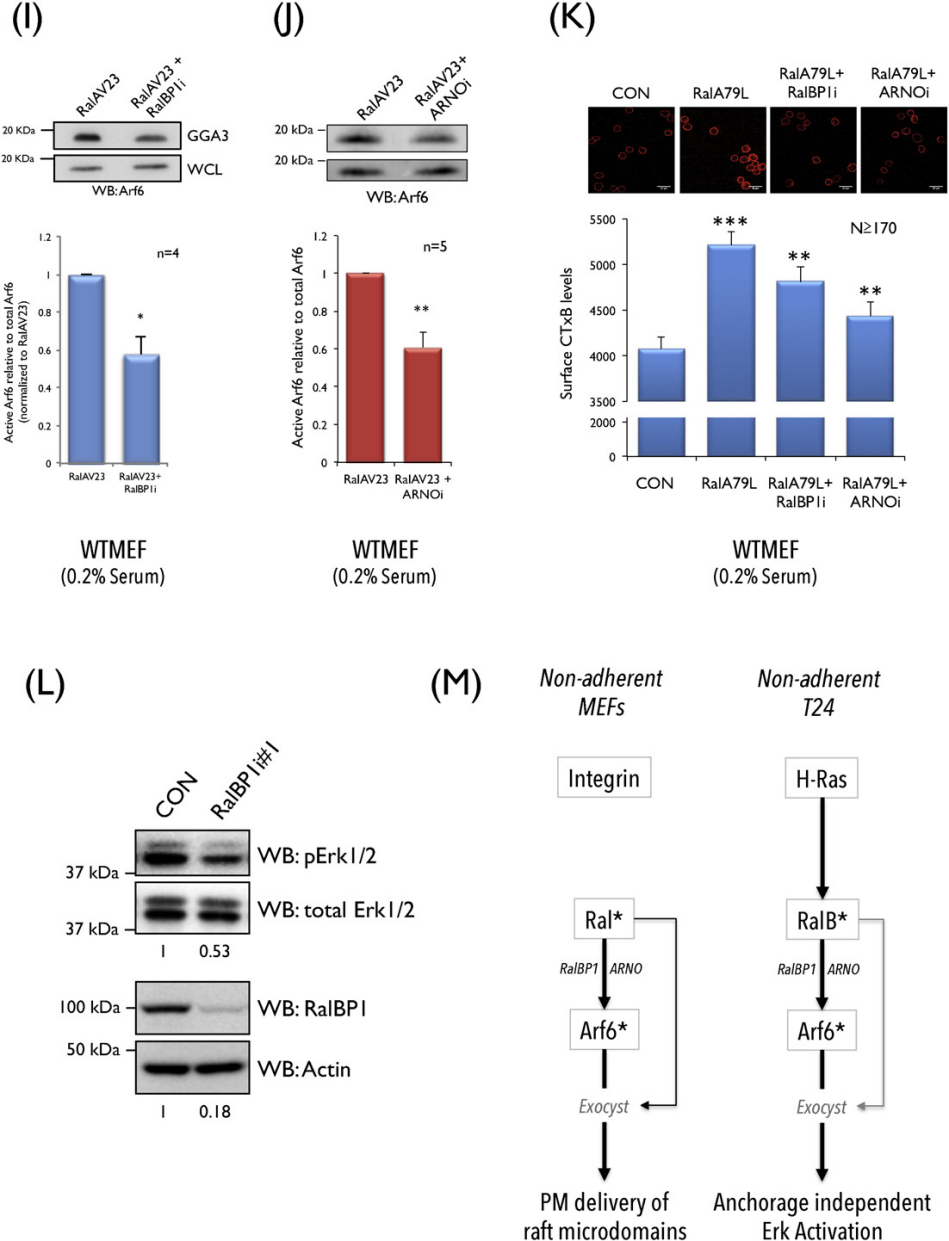
**Role of Ral Effectors in mediating Ral dependent Arf6 activation**. Western blot detection and quantitation of active Arf6 pulled down by GST-GGA3 (GGA3) and total Arf6 in whole cell lysate (WCL) is represented in the following graphs. (**A**, **B**) In low serum control (CON) and (**A**) Sec5 knockdown (Sec5i) and (**B**) RalBP1 knockdown (RalBP1i) MEFs which were stable adherent (SA), suspended (SUSP) or readherent on fibronectin (FN). (**C**) In siRNA resistant FLAG RalBP1* expressing stable adherent (SA) and suspended (SUSP) RalBP1 knockdown MEFs (RalBP1i+RalBP1*). (**D‐**) In suspended control (CON) and RalBP1 knockdown T24 cells (**D**) targeted with siRNA sequence #1 (RalBP1i #1) in low serum and (**E**) with siRNA sequence #1 (RalBP1i #1) with serum and (**F**) sequence #2 (RalBP1i #2) with serum. (G-H). In suspended control (CON) and ARNO knockdown T24 cells targeted with (**G**) siRNA sequence #1 (ARNOi #1) with serum and (**H**) sequence #2 (ARNOi #2) with serum. (**I**, **J**) In suspended active Ral (RalAV23) expressing MEFs in low serum (**I**) with and without RalBP1 (RalV23 + RalBP1i) and (**J**) with and without ARNO (RalV23 + ARNOi). Calculated percentage active Arf6 levels were normalized to respective SA/CON/RalAV23. Graphs represent mean ± standard error from a minimum of three and maximum of five independent experiments. Statistical analysis was done using the two tailed single sample *t*-test and their significance represented (* *p* value < 0.05 and ** *p* value < 0.01). (**K**) Cell surface GM1 in control (CON) and active RalA mutant expressing WTMEFs (RalA79L) lacking RalBP1 (RalA79L + RalBP1i) or ARNO (RalA79L + ARNOi) was detected with CTxB-Alexa 594, imaged (top panel) and surface labeling intensity quantitated by measuring integrated density. Integrated density for a minimum of 170 cells was calculated and mean ± standard error represented in the graph. Graph is representative of two independent experiments that gave similar results. Statistical analysis was done using the two tailed unpaired *t*-test and their significance represented (* *p* value < 0.05, ** *p* value < 0.01, *** *p* value < 0.001). Significance for RalA79L is relative to the CON and for RalA79L + RalBP1i/ARNOi is relative to RalA79L (**L**) Western blot detection of Erk phosphorylation (P-p44/42 MAPK-Thr202/Tyr204) (WB:pErk) and total Erk1/2 (p44/42-MAPK) (WB: total Erk) in whole cell lysates from control (CON) and RalBP1 knockdown (RalBP1i) T24 cells held in suspension with serum for 120 min. Following densitometric scanning band intensity ratio of pErk/total Erk and RalBP1/Actin were calculated and normalized to control (CON). Blots are representative of two independent experiments that gave similar results. (**M**) The left panel schematic represents the RalA-Arf6 pathway in suspended WTMEFs expressing active Ral (RalA*) that through RalBP1 and ARNO drives Arf6 activation (Arf6*) to support the plasma membrane (PM) delivery of raft microdomains through the exocyst complex. The right panel schematic represents how in T24 cells with serum the H-Ras dependent activation of RalB (RalB*) through RalBP1 and ARNO drives Arf6 activation (Arf6*) to support anchorage independent Erk signalling.

We hence tested the role Ral effectors could have in regulating this crosstalk by evaluating the role of Sec5 and RalBP1in MEFs and T24 cells. Sec5, a member of the exocyst complex, is known to be involved in integrin dependent membrane raft trafficking and cell spreading [20], making it an attractive candidate. Knockdown of Sec5 (Fig. S4A) did not affect the adhesion dependent activation of Arf6 in re-adherent MEFs (Fig. 5A). This suggests the role Sec5 has in integrin dependent cell spreading could be limited to the exocyst complex [20]. The second candidate, RalBP1, along with being a canonical Ral effector is also reported to be overexpressed in bladder cancers [41] making it of much interest. Downstream of R-Ras RalBP1 is also seen to regulate Arf6 activation independent of Ral [45]. We hence tested its role downstream of Ral in this pathway. Loss of RalBP1 (Fig. S4B) did not affect the adhesion dependent activation of Arf6, but marginally reduced active Arf6 levels in non-adherent MEFs (Fig. 5B) (~ 35% decrease relative to non-adherent control). Interestingly expression of siRNA resistant RalBP1 mutant in these knockdown MEFs (Fig. S4B, S4C) reversed this drop and sustained Arf6 activation, effectively making it anchorage independent (Fig. 5C). To further evaluate its role, RalBP1 was knocked down in T24 cells using two independent siRNA sequences (Fig. S4D, S4E) and seen to significantly decrease anchorage independent Arf6 activation in low (Fig. 5D) and with serum conditions (Fig. 5E, F). The effect loss of RalBP1 has on Arf6 activation was comparable to loss of RalB (Fig. 3G) and their joint knockdown (RalBP1 + RalB) (data not shown) suggesting their effect to be mediated along a common RalB-RalBP1-Arf6 pathway in T24 cells. RalBP1 is known to bind the Arf6 GEF ARNO (cytohesin-2) to regulate Arf6 activation in mouse fibroblasts [45], [46]. Knockdown of ARNO in T24 cells using two independent siRNA sequences significantly decreased anchorage independent Arf6 activation (Fig. 5G and H, S4F). Knockdown of cytohesin1 and cytohesin3, seen to be expressed as well as cytohesin2 (ARNO) in T24 cells (Fig. S4K), did not affect anchorage independent Arf6 activation (Fig. S4L, S4M) suggesting this regulation of Arf6 to indeed be cytohesin-2 (ARNO) specific. RalBP1 and ARNO we also found did not affect anchorage independent RalA or RalB activation (Fig. S4G, S4H, S4I, S4J) supporting their regulation of Arf6 to be downstream of Ral along a Ral-RalBP1-ARNO-Arf6 pathway in T24 cells.

To confirm this, we tested the effect siRNA mediated knockdown of RalBP1 and ARNO (Cytohesin-2) have downstream of active RalA in mediating Arf6 activation in WTMEFs (Fig. 2A). In active RalA (RalAV23/RalA79L) expressing MEFs (Fig. S4N, S4O, S4P) loss of RalBP1 or ARNO (Fig. S4Q, S4R) significantly decreased anchorage independent Arf6 activation (Fig. 5I and J) and raft microdomain delivery at the plasma membrane (Fig. 5K) confirming their role downstream of Ral. Accordingly loss of RalBP1 is also seen to reduce anchorage independent Erk activation in T24 cells (Fig. 5L). Together this identifies the Ral-RalBP1-ARNO-Arf6 pathway as a mediator of the Ral-Arf6 crosstalk, membrane trafficking and signalling (Fig. 5M).

## 3 Discussion

In defining the regulatory crosstalk between RalA and Arf6 downstream of integrins and oncogenic Ras, this study not only reveals the role Arf6 has downstream of Ral but also how it could help mediate Ral isoform specific function in cells. The differential activation of Ral isoforms by specific stimuli we find contributes to this crosstalk through the differential utilization of a Ral effector (RalBP1) with the Arf6 GEF (ARNO).

Downstream of integrins this crosstalk explains the differential role known for Ral and Arf6 [19], [20] in the exocyst complex mediated delivery of raft microdomains at the plasma membrane. With Ral working upstream of Arf6 (and not vice versa) targeting Arf6 in an active Ral background blocks the delivery of raft micrdomains. This reveals Arf6 and this crosstalk to be necessary for Ral-exocyst mediated function along this pathway [20]. This when considered in context of the fact that active Arf6 by itself cannot mediated this delivery [19], suggests the linear integrin-RalA-Arf6 pathway to be further supported by a role for Ral with active Arf6 in mediating this delivery (Fig. 2D). Such a joint role could be envisaged as part of the exocyst complex. Spatial activation of Arf6 by Ral could facilitate its binding to the exocyst component Sec10 allowing for the tethering and delivery of vesicles at the plasma membrane [24]. Such a localization of Arf6, through its association with Rab11 binding FIP3 and FIP4 is seen to recruit the exocyst complex to the midbody during cytokinesis [47]to which Ral also localizes [6]. Arf6 dependent recruitment and activation of PI(4)P 5-kinase [48] could also generate (PI(4,5)P_2_) at the plasma membrane [49], [50] that the exocyst component Exo70 can bind [51]. With both integrins and active Ras localizing at the plasma membrane [52], [53], [54], like Ral and Arf6 do in re-adherent MEFs (Fig. S1L), this could indeed be the major site where this crosstalk is functional.

While active RalA and RalB can both mediate Arf6 activation, their differential regulation of this crosstalk downstream of specific stimuli could have a role in mediating their isoform specific function in cells. This regulation by individual Ral isoforms is directly dependent on their differential activation by specific stimuli. Downstream of integrins preferential activation of RalA (over RalB) means it drives Arf6 activation to regulate exocyst function and integrin dependent cell spreading [20]. Downstream of oncogenic H-Ras this regulation is further nuanced by the presence or absence of growth factors. In T24 cells with serum preferential activation of RalB (over RalA) means it drives Arf6 activation to differentially regulate anchorage independent Erk signalling in these cells (Fig. 4A, C, and 3G). RalA with its modest effect on Arf6 activation affects this pathway only marginally, unless it is targeted with Arf6. This hence suggests RalA could work as well as RalB along this pathway, but the preferential activation of RalB and its regulation of Arf6 makes it the more prominent mediator of Erk signalling [40], [42], [44]. In non-adherent WTMEFs as well we find RalA and RalB to both be capable of regulating Arf6 (Fig. 2A and B), though in re-adherent cells the preferential activation of RalA and its regulation of Arf6 (Fig. 1A) makes it the primary mediator of cell spreading [20].

While Arf6 is not a direct Ral effector, its activation by active Ral does suggest the possible involvement of a Ral effector(s) in mediating this crosstalk. RalBP1 we find to be such a mediator, regulating anchorage independent Arf6 activation in active Ral expressing MEFs and T24 cells (Fig. 5E, F and I). However it does not regulate the RalA-Arf6 crosstalk in re-adherent MEFs (Fig. 5B). This suggests the Ral-Arf6 crosstalk while conserved could be regulated differently depending on the stimulus. Active Ral could mediate the localization of RalBP1, as seen in the mitochondria [55], to sites of Arf6 activation at the plasma membrane. This inturn could recruit an Arf6 GEF like ARNO (cytohesin-2), known to bind RalBP1 [45], [56], to the site of Ral and Arf6 activation. Functionally, RalBP1 mediated regulation of Arf6 downstream of Ral does affect the plasma membrane delivery of raft microdomains in WTMEFs (Fig. 5K) and anchorage independent Erk signalling in T24 bladder cancer cells (Fig. 5L).

While this study has focused on understanding the Ral-Arf6 crosstalk in WTMEFs and bladder cancer T24 cells (expressing H-Ras), we also detected Ral independent activation of Arf6 in pancreatic cancer MiaPaCa2 cells (expressing K-Ras). This hence suggests the crosstalk could be cell type and/or stimuli specific. Alternate mechanism(s), such as overexpression of Arf6 or Arf6 GEFs [57], [58], [59], [60] could mediate Ral independent Arf6 activation. The differential association between Ral and Arf6 in H-Ras vs K-Ras backgrounds, as suggested in their regulation of Phospholipase D1 (PLD1) [25], could further contribute to this difference. It does however raise the question if this difference in how Arf6 is regulated in these two cell lines is conserved across other H-Ras and K-Ras dependent cancers. The contribution Arf6 makes to Ral dependent growth signalling in these cancers could also be different. Beyond their role in membrane trafficking and anchorage independent signalling, Ral and Arf6 are involved in regulating several other cellular pathways including PLD1 activation [25], [26], GLUT4 receptor trafficking [61], [62], cellular cytokinesis [6], [47], insulin secretion [27], [31], [32] and Fc-gamma-R mediated phagocytosis [33], [34]. The stimulus specific nature of the Ral-Arf6 crosstalk, its regulation and contribution along these pathways will be worth exploring.

## 4 Materials and methods

### 4.1 Reagents

Human Plasma fibronectin (Catalog #F2006) and 3X FLAG peptide (F4799) were procured from Sigma. Cholera toxin subunit B (CTxB) labelled with Alexa 594 (C22843) was from Molecular Probes and used at a 1:5000 dilution. Antibodies used include Anti RalA (BD Transduction) (CLONE 8, Catalog No. 610221) at a 1:2000 dilution [20], Anti RalB (R&D Biosystems) (catalog No. AF3204, Batch No. W1S01) at a 1:1000 dilution [20], Anti Arf6 (gift from Dr. James Casanova) at 1:2000 dilution [19], Anti-FLAG (Sigma) (Clone M2, Catalog No. F3165, Batch No. SLBH1191V) at 1:5000 dilution and Anti-FLAG-HRP (Sigma) (Clone M2, Catalog No. A8592) at 1:2000 dilution, Anti β tubulin (Developmental Studies Hybridoma Bank) (Clone E7, Catalog No. AB_2315513) at 1:2000 dilution, Anti RalBP1 (Cell Signalling Technology)(Clone I33, Catalog No 3630S, batch No. 0001) at 1:1000 dilution [63], Anti pErk1/2 (P-p44/42 MAPK-Thr202/Tyr204) (Cell Signalling) (Cat# 9101 Lot# 23) at 1:2000 dilution and anti Erk1/2 (p44/42-MAPK) (Cell Signalling) (Cat # 9102 Lot# 26) at 1:1000 dilution. Anti-β Actin (Abcam)(Clone ACTN05(C4), Catalog No. ab3280) at 1:2000 dilution, anti Sec5 (N15) (Santa Cruz Biotechnology) (Clone N-15, Catalog No. sc-30285, Batch No. D2809) at 1:500 dilution [20]and Anti-HA (Roche) (Clone 3F10, Catalog No. 11867423001) at 1:1000 dilution. Published RNA interference sequences used in this study were procured from Sigma (see supplementary text for detailed listing). SYBR Safe DNA gel stain was obtained from Invitrogen (Catalog No. S33102). On-target Plus siRNA smartpool against mouseSec5 (L-042601), mouseARNO (L-059077) and humanARNO (L-011925) were from Dharmacon (sequence listing in supplementary text). FLAG-WT-RalA, FLAG-G23V-RalA, untagged G23V-RalA, FLAG-G23V-RalB were kind gifts from Dr. Michael White's lab and pSuper-shArf6-Neo-GFP from Dr. Eunjoon Kim's lab [64]. CFP-RalA-WT, CFP-RalA-V23, HA-RalB-WT and YFP-RalB-V23 were kind gifts from Dr. Dan Theodorecu's lab. HA-H-Ras-GV12 was kind gift from Dr. John Hancock's lab [65].

### 4.2 Tissue culture

Mouse embryonic fibroblasts (MEFs)(from the lab of Dr. Richard Anderson, University of Texas Health Sciences Center, Dallas TX) were cultured in high glucose DMEM medium with 5% fetal bovine serum (FBS). MiaPaCa2 and T24 cells were procured from ECACC for these studies and were cultured in RPMI1640 and high glucose DMEM respectively with 5% FBS. HEK293T cells were procured from ATCC and were cultured in high glucose DMEM with 5% FBS and 1 × PenStrep. Original vial of cells were grown and frozen down and re-thawed during the course of experiments. Cells were regularly checked for and found to be without any bacterial or mycoplasma contamination. Transfections were done in 6 well plate or 60 mm dishes with 2 μg or 5 μg of plasmid DNA using the Lipofectamine LTX (Invitrogen) reagent. For knockdowns, cells seeded in 60 mm dishes were transfected with 100 pmol duplex siRNA oligo using the RNAiMax transfection reagent (Invitrogen). This was similarly repeated the next day and cells used 48 hours later. For reconstitutions, rescue vectors were electroporated (for RalA) (30 μg plasmid + 10 μg salmon sperm DNA), or transfected with LTX (for RalBP1) (12 μg) 24 h after second siRNA transfection, allowed to recover for 24 h and then used. Stable shArf6 MEFs were selected with G418 (Roche) after transfection with pSuper-shArf6-Neo-GFP. Serum starvation, suspension and replating of cells was done as earlier [19], [20]. For combined siRNA and plasmid transfections 120 pmol of siRNA and 4 μg of plasmid DNA were transfected using Lipofectamine 2000 transfection reagent (Invitrogen) and cells were used 48 h post transfection.

### 4.3 RNA isolation and RTPCR

Total RNA was isolated using Trizol reagent (Invitrogen) and cDNA prepared using Reverse Transcriptase and Oligo-dT primers (Promega). Quantitative PCR done using SYBR FAST qPCR master mix (Kapa Biosystems) in BioRad CFX96 Real Time System. Primers used are listed in supplementary text.

### 4.4 Plasmids and site directed mutagenesis

FLAG R79L RalA mutant was made by site-directed mutagenesis of FLAG WT RalA and FLAG RalBP1* (siRNA insensitive mutant) by site-directed mutagenesis of FLAG WT RalBP1 (gift from the Dr. Lawerence Goldfinger lab). Primers designed using QuikChange tool (Agilent Technologies) are listed in supplementary text. The siRNA insensitive HA-RalA* mutant is as described earlier [19], [20].

### 4.5 Cell surface labeling with CTxB

MEFs grown in low serum conditions (0.2% serum) were held in suspension labelled and mounted as described earlier [20]. They were imaged simultaneously using identical setting with a Zeiss LSM 710 laser confocal-Anisotropy microscope with a 40 × oil objective and analyzed using the Image J software (NIH). Thresholds were used to define cell edge and create a mask. Total integrated density in cells was measured and average integrated density calculated.

### 4.6 Arf6 and Ral activity assay

Cells with serum (5% FBS) or low serum (0.2% FBS) were held in suspension (Susp), replated on fibronectin (10 μg/ml) for 15 min (FN) or stable adherent for 4 h (SA). Cells were lysed and processed for Arf6 activity [19] or Ral activity [20] as described earlier. Blots were developed using the LAS4000 detection system (Fujifilm-GE) and densitometric band analysis done using Image J software (NIH). Percent active levels of Arf6 or RalA/RalB were determined by using the following calculation.

$$\text{Percentage Activity}=\frac{\text{Pulldown Band Intensity}\;x\;100}{\text{Corresponding}\;\mathrm{WCL}\;\text{band intensity}\;x\;\text{Dilution Factor}}$$

The dilution factor was calculated as the ratio of the amount of total cell lysate used for the pulldown (400 μl) and the amount of this lysate resolved by SDS PAGE in the whole cell lysate (WCL) lane (22.5 μl WCL + 7.5 μl 4 × Lamellis). The dilution factor was hence 400 ÷ 22.5 = 17.77. This ratio was kept constant in all our experiments. Active Arf6 or active RalA levels under different treatment conditions were normalized to stable adherent (SA) or control (CON).

### 4.7 Co-immunoprecipitation studies

HEK293T cells transfected with 7 μg of FLAG/FLAG-WT-RalA/FLAG-G23V-RalA/FLAG-WT-RalB/FLAG-G23V-RalB and 7 μg of HA-T157A-Arf6 with Lipofectamine LTX reagent for 48 h were lysed (20 mM HEPES pH 7.5, 400 mM KCl, 5% Glycerol, 5 mM EDTA, 0.4% NP40 + 1X PIC + phosphatase inhibitors) for 30 min on ice, sonicated and spun down at 15,000 rpm for 30 min. Lysates (700 μg equivalent) were incubated with Protein G-Dyna beads (Invitrogen) bound to Anti-FLAG (M2) antibody for 30 min at 4 °C on a rotary mixer in binding buffer (20 mM HEPES pH 7.5, 50 mM KCl, 5% Glycerol, 5 mM EDTA, 0.05% NP40 + 1X PIC + phosphatase inhibitors) [66]. The immune-complexes were washed and eluted with 200 μg/ml of 3 × FLAG peptide. Immunoprecipitates and whole cell lysates (WCL) were probed with anti-HA and anti FLAG-HRP antibodies and blots developed using the LAS4000 detection system (Fujifilm-GE).

### 4.8 Immunofluorescence studies

MEFs transfected with 2 μg of CFP-WT-RalA/CFP-G23V-RalA/HA-WT-RalB/YFP-V23-RalB and 2 μg of FLAG-T157A-Arf6 using Lipofectamine LTX reagent were trypsinized, washed and replated on fibronectin (10 μg/ml) for 15 min. Following fixation (3.5% paraformaldehyde) and permeabilization (PBS containing 0.1% Triton-X-100 - 5 min), cells were blocked with 10% BSA for 30 min at 37 °C, incubated with 1:2000 anti-FLAG (M2) and anti-HA antibody (Roche) in 3% BSA for 1 h at 37 °C. Cells were finally stained with 1:1000 diluted secondary antibody for 1 h at room temperature. All incubations were done in a humidified chamber. Stained coverslips were washed and mounted with Fluoromount-G (Southern Biotech) and imaged using a Zeiss LSM 710 laser confocal-Anisotropy microscope with a 63 × oil objective.

### 4.9 Anchorage independent Erk signalling in T24 cells

T24 cells in 60 mm dishes were treated twice with 50 pmol of each duplex siRNA oligo (hRalA/hRalB/hArf6-1 + hArf6-2/hRalBP1) or in combination (hRalA + hArf6-1 + hArf6-2/hRalB + hArf6-1 + hArf6-2) using RNAiMax transfection reagent (Invitrogen) as described earlier. 60 h after the second transfection cells were detached held in suspension for 120 min with 5% serum [20]and then lysed. Cell equivalent amount of lysates resolved by SDS PAGE and western blotted were probed with pErk (Thr 202/Tyr 204) and total Erk antibodies. Blots were developed using chemiluminescent substrates (Pierce) with the LAS 4000 developing system (Fujifilm-GE). Densitometric analyses of band intensities were done using Image J software (NIH) and pErk levels were normalized to total Erk.

### 4.10 Statistical analysis

Statistical analysis of data was done using the two tailed unpaired Student's *t*-test and when normalized to respective controls using the two tailed single sample *t*-test. All analysis was done using Prism Graphpad analysis software.

## Author contributions

***Archana Pawar*** – Experimental Design, Execution of Experiments, Data Analysis, Writing of Manuscript.

***Jeremy A***. ***Meier*** – Experimental Design, Execution of Experiments, Data Analysis.

***Anwesha Dasgupta*** – Experimental Design, Execution of Experiments, Data Analysis.

***Neha Diwanji*** – Experimental Design, Execution of Experiments, Data Analysis.

***Neha Deshpande*** - Experimental Design, Execution of Experiments, Data Analysis.

***Kritika Saxena*** – Experimental Design, Execution of Experiments, Data Analysis.

***Natasha Buwa*** – Experimental Design, Execution of Experiments, Data Analysis.

***Siddhi Inchanalkar*** – Experimental Design, Execution of Experiments, Data Analysis.

***Martin Alexander Schwartz*** – Experimental Design, Writing of Manuscript.

***Nagaraj Balasubramanian*** – Experimental Design, Execution of Experiments, Data Analysis, Writing of Manuscript.

## Funding

This work is funded by a grant from the Wellcome Trust DBT India Alliance for NB WT_DBT_30711059 and USPHS grant RO1 GM47214 for MAS. AP and Natasha Buwa are supported by a fellowship from the Council of Scientific and Industrial Research (CSIR), India. SI is supported by a fellowship from the Department of Biotechnology (DBT), India.

## Appendix A Supplementary data

**Supplementary Fig**. **S1**: Representative blot showing knockdown of (**A**) RalA, (**B**) RalB and (**C**) Arf6 relative to tubulin (WB: Tubulin). (**D**) Representative western blot showing the detection of endogenous RalA in RalA knockdown MEFs (RalAi) expressing HA tagged hRalA* mutant resistant to siRNA (indicated by arrow) (RalAi + hRalA*). Western blot detection of (**E**) RalA and (**F**) RalB to confirm the expression of untagged RalA-V23 (WB: RalA) and FLAG-RalBV23 (WB: RalB)(indicated by arrow) respectively. (**G**, **H**) Western blot detection of active Ral pulled down with GST-Sec5 (Sec5 PD) from whole cell lysate (WCL) of cells expressing (**G**) untagged active RalA V23 (WB: RalA), (**H**) FLAG tagged wild type RalB (RalB WT) and active RalBV23 (RalB V23) (WB: Flag) confirms their activation status. (**I**) Stable expression of pSuper-shArf6-GFP-Neo plasmid in wild type MEFs detected by GFP fluorescence (GFP) is seen in all cells detected by phase contrast imaging (Phase). Western blot detection of Arf6 (WB: Arf6), RalA (WB: RalA), Caveolin-1 (WB: Cav-1) and β-tubulin (WB: tubulin) in control (CON) and shArf6 knockdown MEFs (shArf6) confirms Arf6 knockdown without affecting other proteins. (**J**) Cell surface GM1 bound CTxB-Alexa 594 was imaged from suspended control (WTMEF) and GFP shArf6 expressing MEFs (shArf6 MEF) (left panel). Intensity of labeling was quantitated by measuring integrated density for a minimum of 50 cells, calculating their mean and normalizing the same in shArf6 MEF to their respective control (WTMEF). Graph represents mean ± standard error from 3 experiments. (**K**) FLAG immunoprecipitate from empty FLAG, FLAG-WT and FLAG-V23 RalA (top panel) and RalB (lower panel) expressing HEK293T cells with HA-T157Arf6 were western blotted for FLAG (WB: FLAG-HRP) and Arf6 (WB: HA). Blots are representative of three independent experiments. (**L**) Representative confocal images showing the colocalization at membrane ruffles in readherent MEFs of CFP tagged WT RalA (CFP-RalA WT) and V23 RalA (CFP-RalA V23) and HA tagged WT RalB (HA-RalB WT) and YFP tagged V23 RalB (YFP-RalB V23) with FLAG tagged active T157A Arf6 (FLAG-T157A Arf6), detected with anti-HA and anti-FLAG antibodies respectively. Images are representative of two independent experiments (**M**) Whole cell lysates from MEFs expressing WT RalA (CFP-RalA WT) or V23 RalA (CFP-RalA V23) with FLAG tagged active T157A Arf6 (FLAG-T157A Arf6) were probed with anti-RalA (WB: RalA), anti-Arf6 (WB:Arf6) and anti-FLAG (WB: FLAG) antibody. Whole cell lysates from MEFs expressing HA tagged WT RalB (HA-RalB WT) or YFP tagged V23 RalB (YFP-RalB V23) with FLAG tagged active T157A Arf6 (FLAG-T157A Arf6) were probed with anti-RalB(WB: RalB), anti-Arf6 (WB:Arf6) and anti-FLAG (WB: FLAG) antibody. Bands representing the expressed and endogenous protein probed for are marked by an arrow on the side of the blots. **Supplementary Fig**. **S2**: Western blot detection and quantitation of active RalA/RalB pulled down by GST-Sec5 (Sec5 PD) and total RalA/RalB in respective whole cell lysates (WCL) from (**A**) MiaPaCa2 cells with low serum (**B**) (**C**) MiaPaCa2 cells with normal serum and (**G**) T24 cells with low serum that are stable adherent (SA) or held in suspension for 120 min (SUSP). Calculated percentage active RalA/RalB levels were normalized to respective SA. Graphs represent mean ± standard error from minimum of three and maximum of four independent experiments. Western blot detection and quantitation of active Arf6 pulled down by GST-GGA3 (GGA3 PD) and total Arf6 in respective whole cell lysates (WCL) from (**D**) MiaPaCa2 cells with normal serum that are stable adherent (SA) or held in suspension for 120 min (SUSP). Calculated percentage active Arf6 levels were normalized to respective SA. Graph represents mean ± standard error from four independent experiments. (**E**, **F**, **H**, **I**) Western blot detection and quantitation of RalA (WB:RalA) or RalB (WB:RalB) relative to tubulin (WB:tubulin) or actin (WB:actin) in MiaPaCa2 cells with low serum (**E**) with and without RalA (RalAi) or (**F**) RalB (RalBi). Similar expression of RalA and RalB was detected and quantitated in T24 cells with and without RalA (RalAi) or RalB (RalBi) in (**H**) low serum or (**I**) normal serum conditions. Graphs represent mean ± standard error for expression in knockdowns relative to their respective control (CON) from a minimum of three and maximum of four independent experiments as indicated in the graph. (**J**) Blot is a representative western blot of whole cell lysates from control (CON) and HA tagged oncogenic H-Ras (H-Ras G12V) expressing cells probed with anti-HA (WB: HA) and actin (WB: actin) as loading control. **Supplementary Fig**. **S3**: Representative western blots and quantitation of (**A**) RalA (WB:RalA), (**B**) RalB (WB:RalB) and (**C**) Arf6 (WB:Arf6) levels relative to actin (WB: Actin) in control (CNT), RalA knockdown (RalAi), RalB knockdown (RalBi), Arf6 knockdown (Arf6i), RalA and Arf6 knockdown (RalAi + Arf6i), RalB and Arf6 knockdown (RalBi + Arf6i) T24 cells with serum. Data was normalized to respective control (CNT). Graphs represent mean ± standard error from four independent experiments. **Supplementary Fig**. **S4**: Representative western blots and quantitation for knockdown of (**A**) Sec5 (Sec5i), (**B**) RalBP1 (RalBP1i) in WTMEFs with low serum, (**D**) RalBP1 with siRNA sequence #1 (RalBP1i #1) in T24 cells with low serum, (**E**) RalBP1 with siRNA sequence #1 (RalBP1i #1) and siRNA sequence #2 (RalBP1i #2) in T24 cells with normal serum. (**F**) Quantitative RTPCR detection and quantitation of the knockdown of ARNO with siRNA sequence #1 (ARNOi #1) and siRNA sequence #2 (ARNOi #2) in T24 cells with normal serum. Graphs represent mean ± standard error relative to respective control (CON) from a minimum of three and maximum of four independent experiments as indicated in the graph. (**C**) Expression of 8 μg and 12 μg of siRNA resistant FLAG tagged RalBP1 (FLAG RalBP1*) in RalBP1 knockdown WTMEFs (RalBP1i) is compared to endogenous RalBP1 levels in control cells (CON). Ratio of the densitometric intensity of RalBP1 (WB: RalBP1) to tubulin (WB: Tubulin) was calculated and normalized to the untransfected control (CON). This is represented in the graph as mean ± standard error from four independent experiments. (**G**–**H**) Western blot detection and quantitation of active RalA/RalB pulled down by GST-Sec5 (Sec5 PD) and total RalA/RalB in respective whole cell lysates (WCL) from control and (**G**) T24 cells with serum where RalBP1 is knocked down with siRNA sequence #1 (RalBP1i #1) or siRNA sequence #2 (RalBP1i #2) or (**H**) ARNO is knocked down with siRNA sequence #1 (ARNOi #1) or siRNA sequence #2 (ARNOi #2). Calculated percentage active RalA/RalB levels in knockdown cells were normalized to their respective control (CON). Graphs represent mean ± standard error from four independent experiments. (**I**) Representative western blots and quantitation for knockdown of RalBP1 with siRNA sequence #1 (RalBP1i #1) or siRNA sequence #2 (RalBP1i #2) in T24 cells with normal serum used in above study. (**J**) Quantitative RTPCR detection and quantitation of the knockdown of ARNO with siRNA sequence #1 (ARNOi #1) and siRNA sequence #2 (ARNOi #2) in T24 cells with normal serum. Graphs represent mean ± standard error relative to respective control (CON) from four independent experiments. (**K**) Quantitative RTPCR was used to detect the expression of Arf6 GEFs cytohesin-1 (CYTH1), cytohesin-2 (CYTH2), cytohesin-3 (CYTH3) and cytohesin-4 (CYTH4) in T24 cells with serum. Delta Ct values for each GEF were calculated relative to actin. Graph represents mean ± standard error for Delta Ct values calculated for each GEF from 3 independent RNA samples. RTPCR samples run on agarose gel and detected using SYBRSafe were used to confirm the expected amplicon size for each GEF RTPCR product. Amplicon sizes are as indicated below each band. (**L**–**M**) Western blot detection and quantitation of active Arf6 pulled down by GST-GGA3 (GGA3) and total Arf6 in whole cell lysate (WCL) from control (CON), (**L**) cytohesin-1 knockdown (CYTH1i) and (**M**) cytohesin-3 knockdown (CYTH3i) T24 cells with serum. Calculated percentage active Arf6 levels were normalized to respective CON. Graphs represent mean ± standard error from a minimum of five and maximum of eight independent experiments. Quantitative RTPCR (qRTPCR) detection and quantitation of the knockdown of cytohesin-1 (CYTH1i) and cytohesin-3 (CYTH3i) is represented in the graph next to activity assay quantitation. Graphs represent mean ± standard error relative to respective control (CON) from a minimum of five and maximum of eight independent experiments. Statistical analysis was done using the two tailed single sample *t*-test and their significance represented (* *p* value < 0.05 and ** *p* value < 0.01). (**N**–**O**) Expression of untagged active RalA (RalAV23) relative to control (CON) in RalBP1 lacking (RalAV23 + RalBP1i) and ARNO lacking (RalAV23 + ARNOi) MEFs is confirmed by detection of RalA (WB:RalA). Equal loading of samples is confirmed by detecting tubulin (WB:Tubulin) or actin (WB:Actin). (**Q**) Representative western blots and quantitation for knockdown of RalBP1 (RalBP1i) in active RalA (RalAV23) expressing WTMEFs is shown in the graph. (**R**) Graph represents RTPCR detection of ARNO in MEFs expressing active RalA (RalAV23) with or without ARNO (RalAV23 + ARNOi). Data from these experiments is normalized to respective CON/RalAV23 and plotted as mean ± standard error from minimum of four and maximum of five independent experiments. **(P)** Expression of FLAG tagged active RalA (RalA79L) relative to control (CON) in RalBP1 lacking (RalA79L + RalBP1i) and ARNO lacking (RalA79L + ARNOi) MEFs is confirmed by detection of RalA (WB:RalA) and comparing the same to actin (WB:Actin). Arrow marks the band corresponding to FLAG tagged RalA79L. Image 1

Supplementary sequence information. Image 2
