# Supplementary material for: Ral-Arf6 crosstalk regulates Ral dependent exocyst trafficking and anchorage independent growth signalling
Source: Cell Signal. 2016 Sep;28(9):1225–36. doi: 10.1016/j.cellsig.2016.05.023 (PMC4973806; doi:10.1016/j.cellsig.2016.05.023)

Supplementary Figure S1 : Pawar et. al.

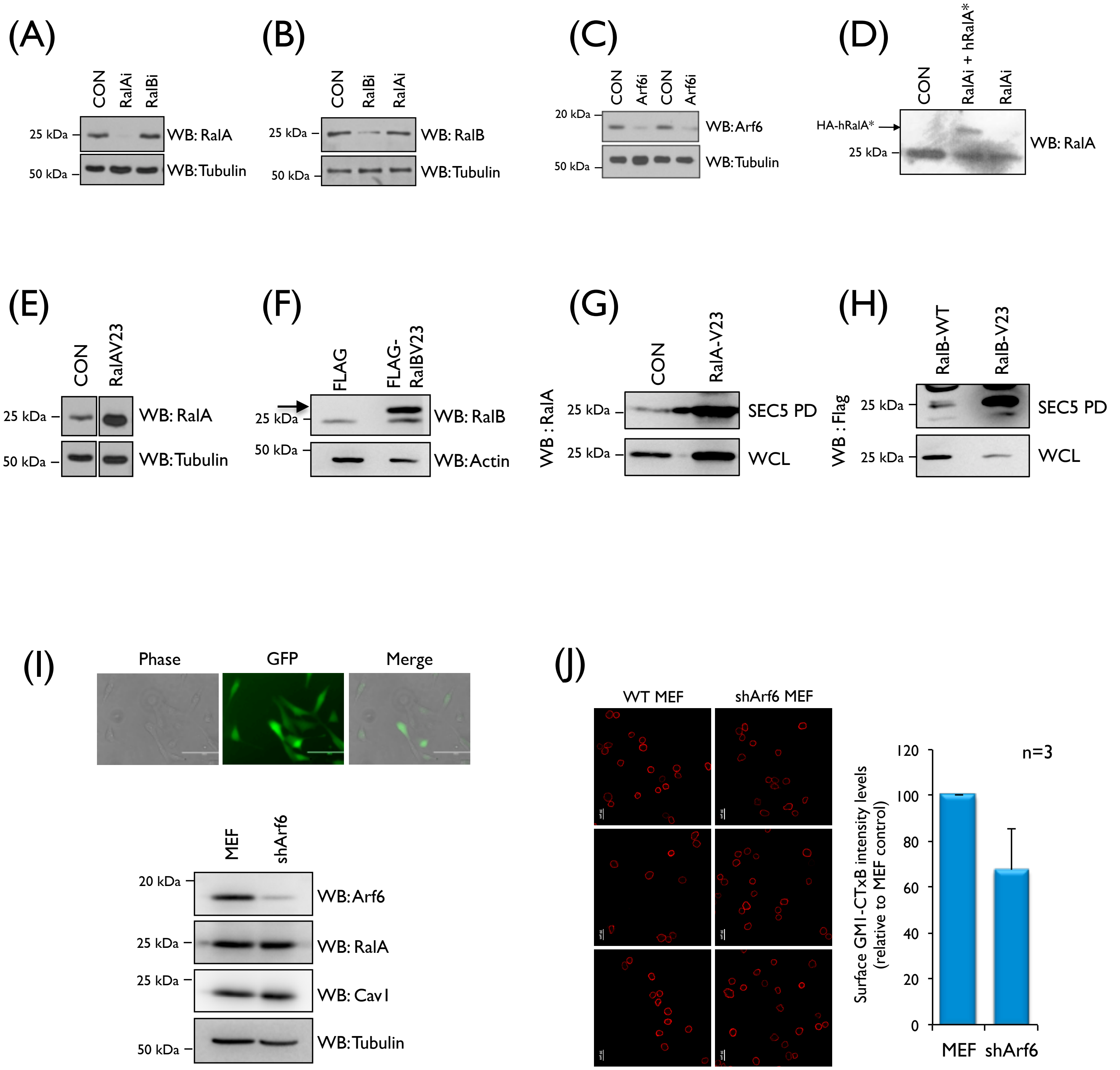

Supplementary Figure S1 : Pawar et. al.

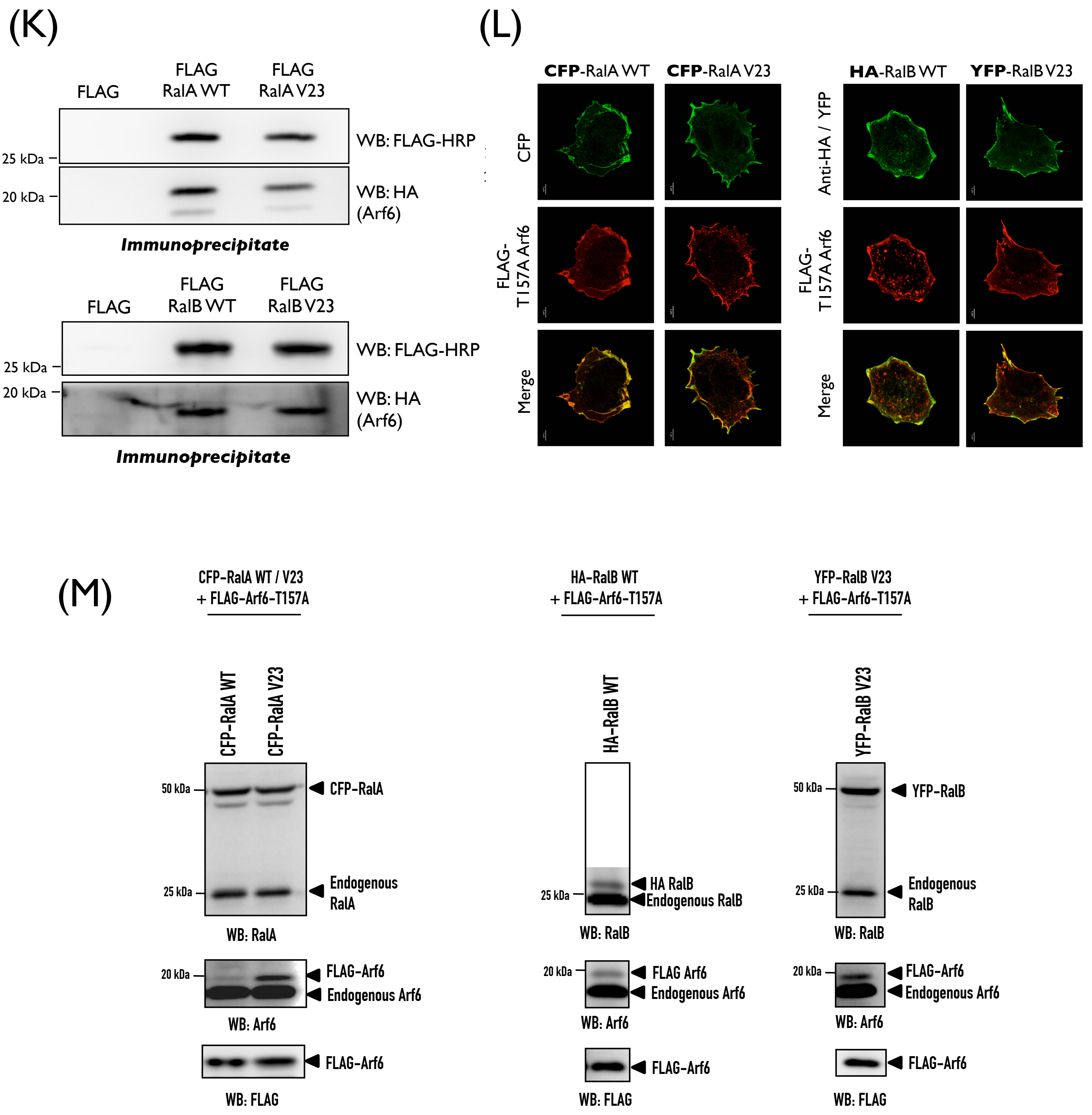

Supplementary Figure S2 : Pawar et. al.

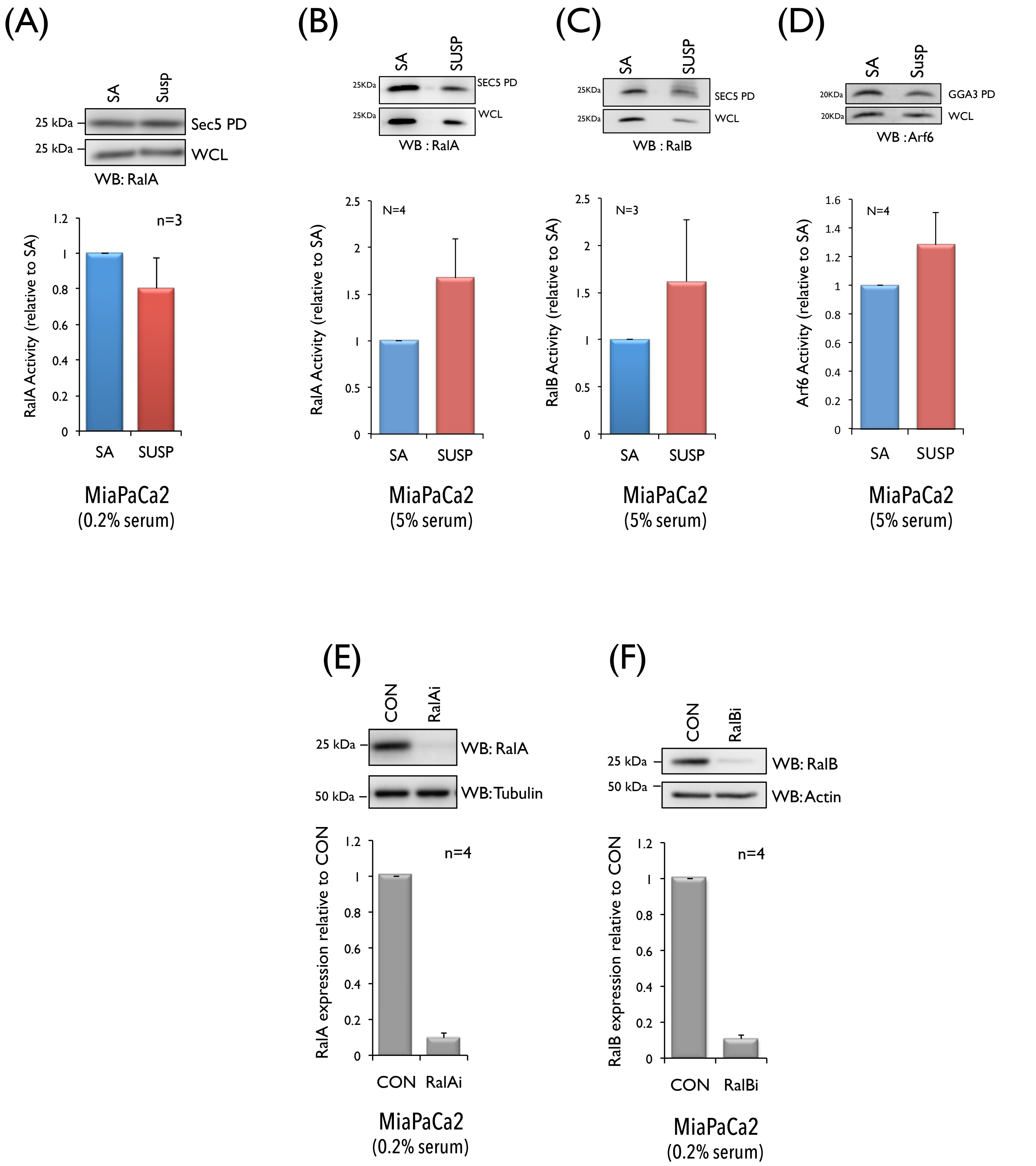

Supplementary Figure S2 : Pawar et. al.

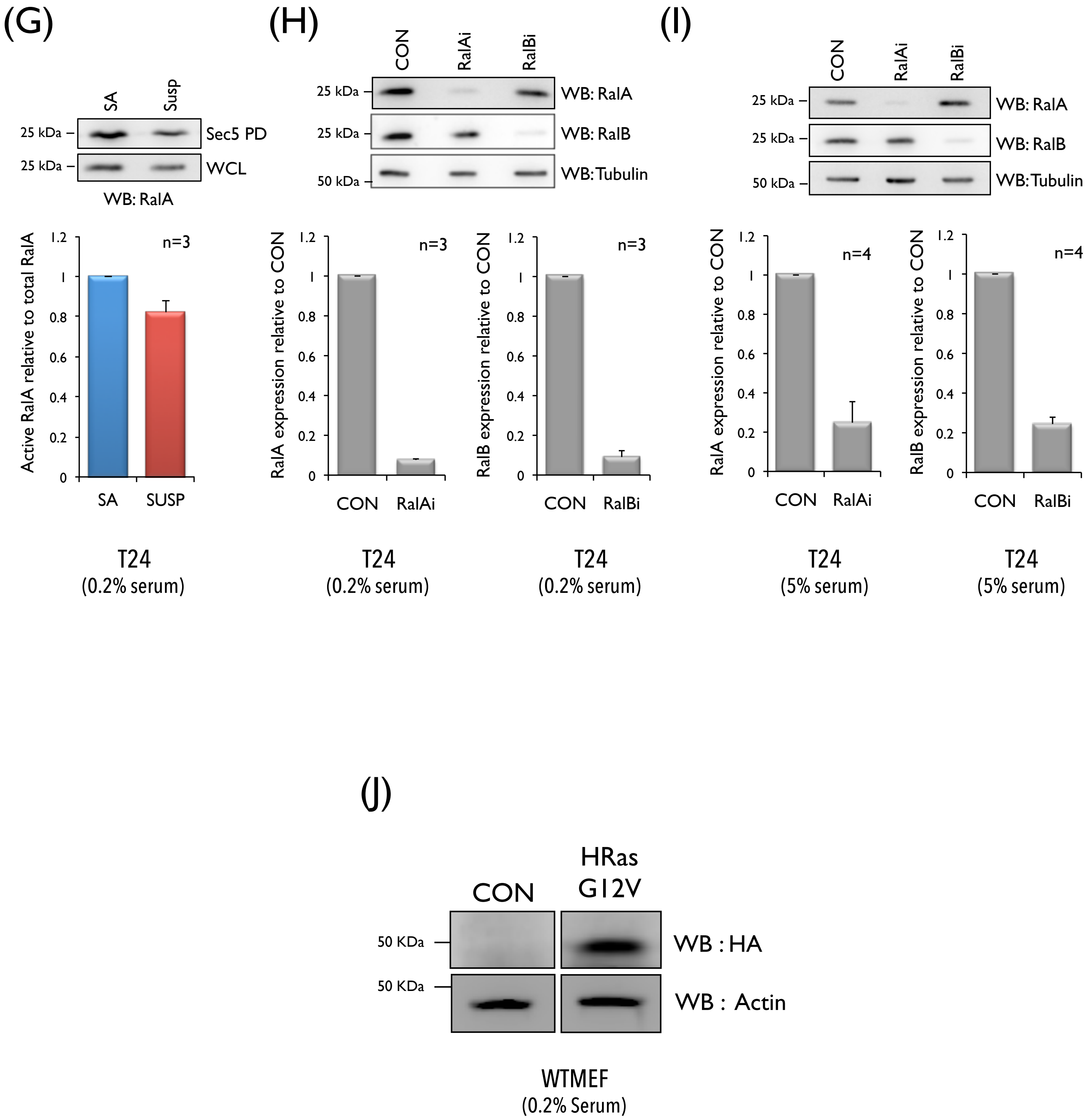

Supplementary Figure S3 : Pawar et. al.

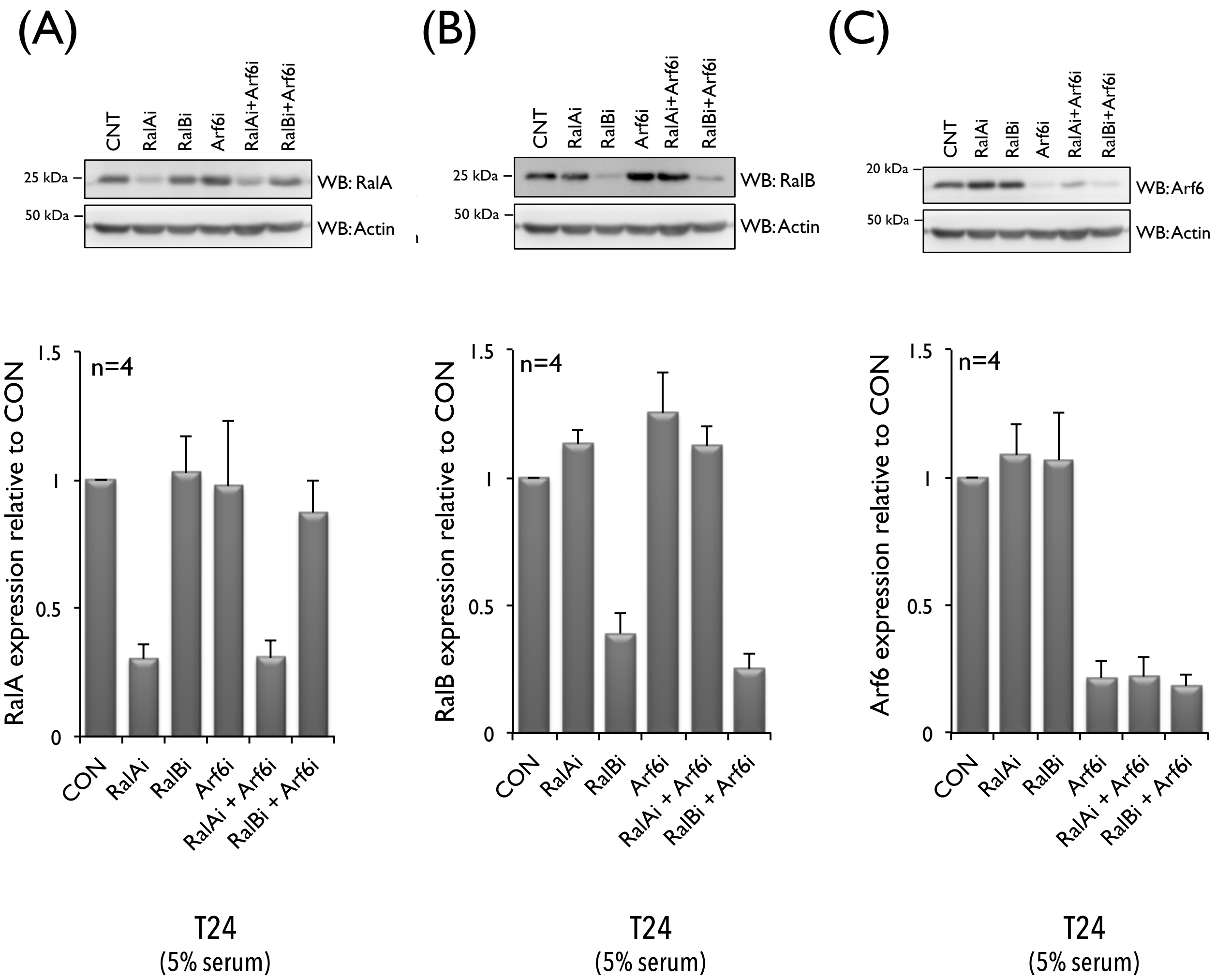

Supplementary Figure S4 : Pawar et. al.

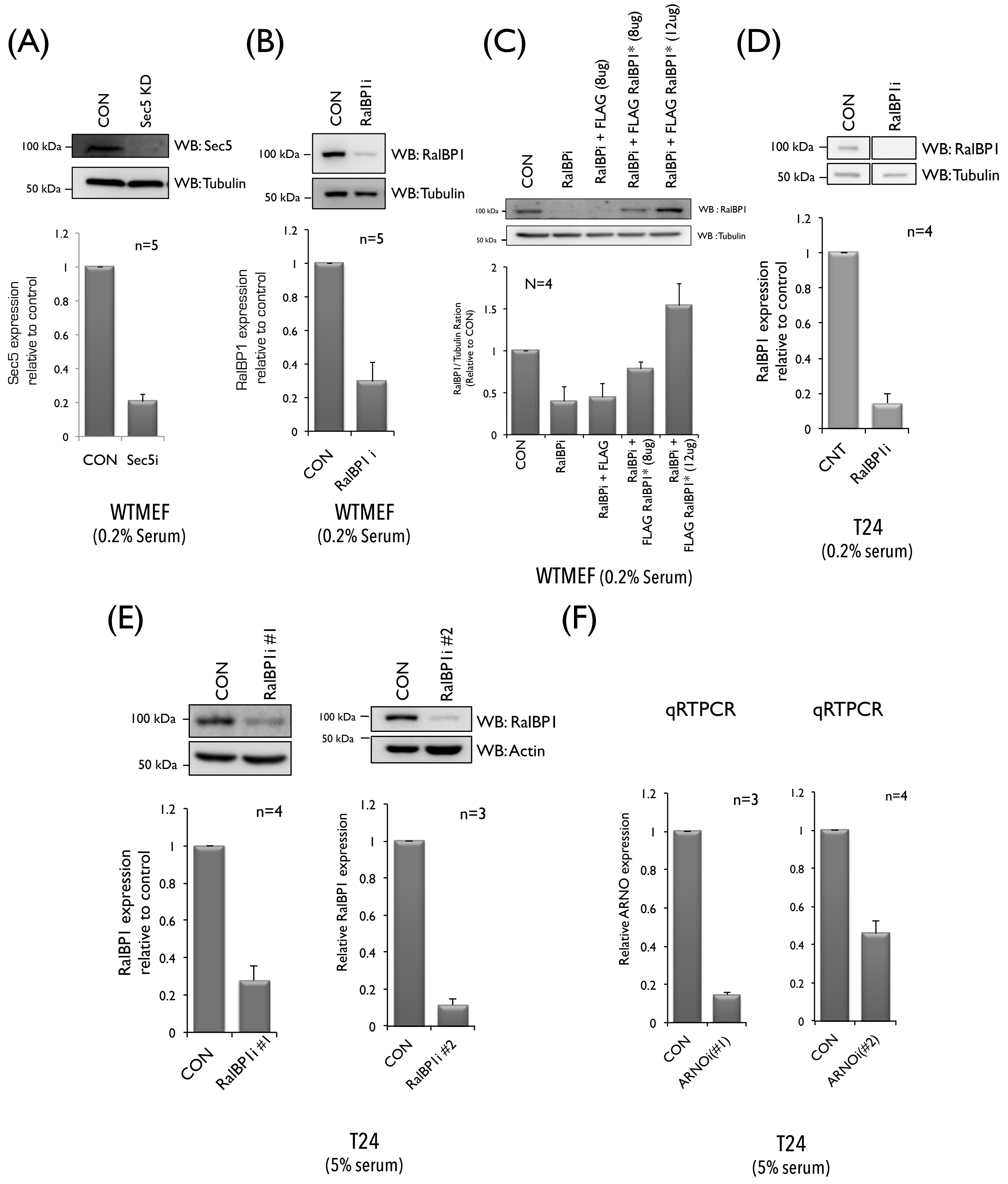

Supplementary Figure S4 : Pawar et. al.

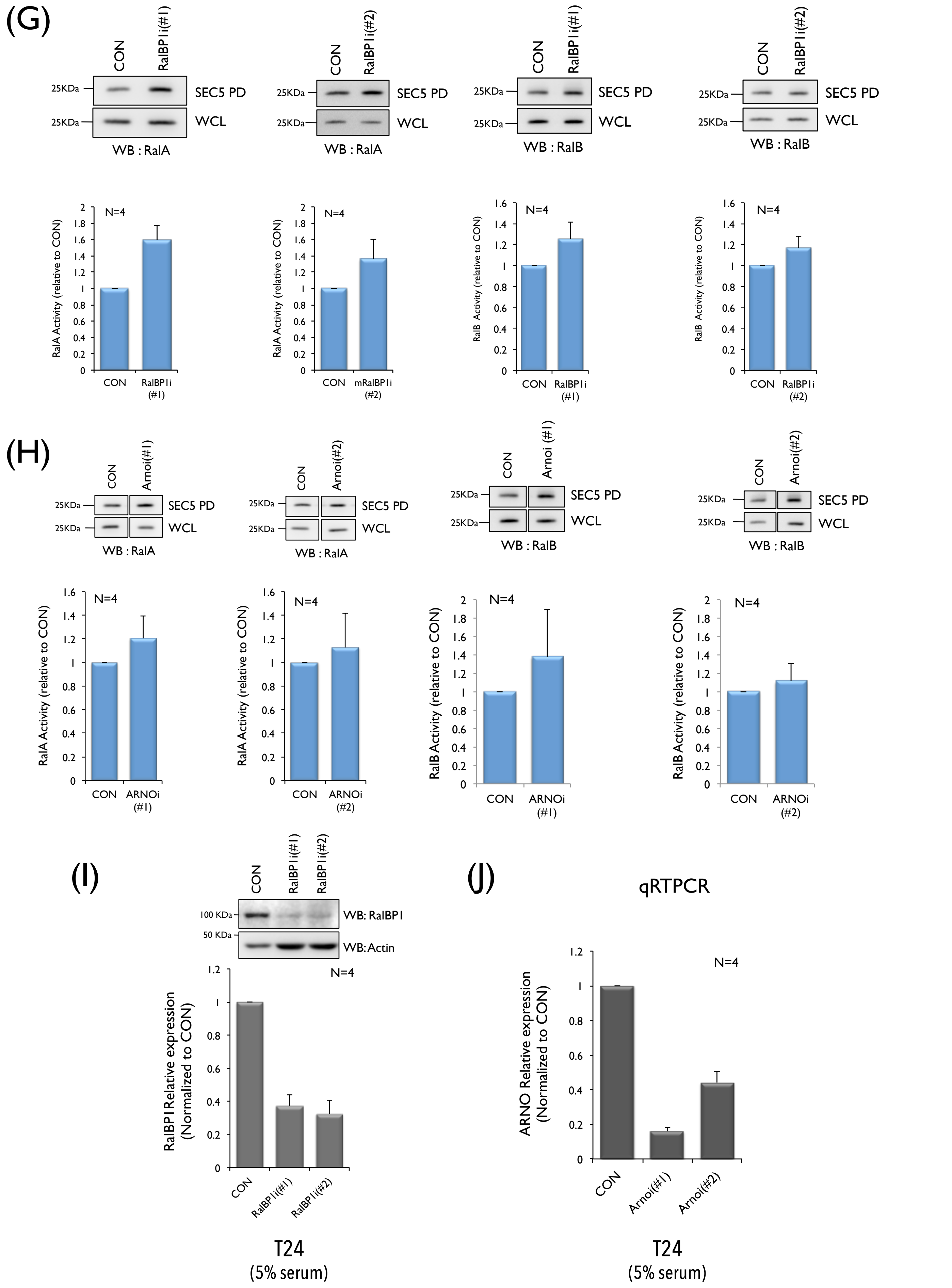

Supplementary Figure S4 : Pawar et. al.

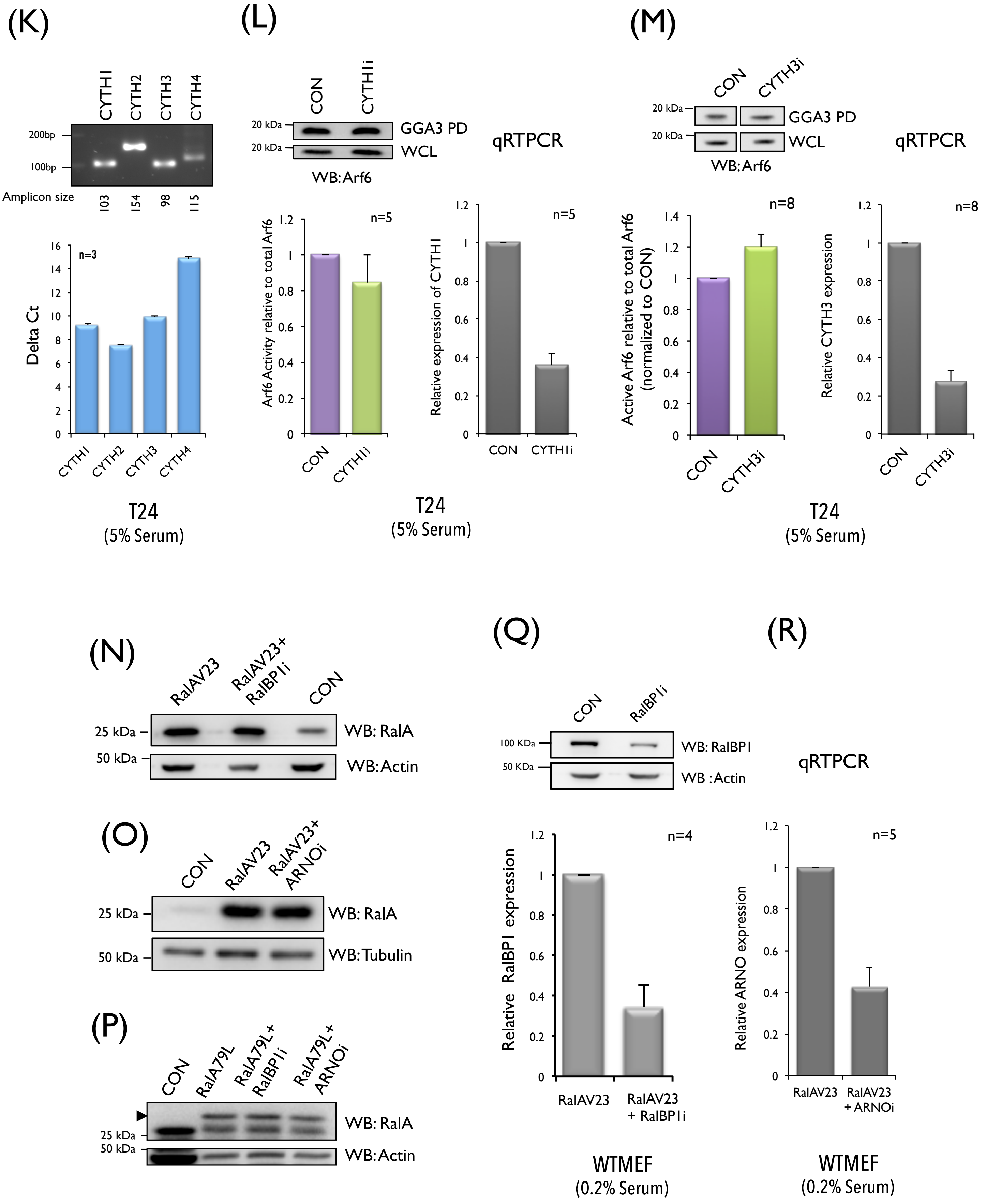

Supplement: Supplementary file 1 — Supplementary Fig. S1: Representative blot showing knockdown of (A) RalA, (B) RalB and (C) Arf6 relative to tubulin (WB: Tubulin). (D) Representative western blot showing the detection of endogenous RalA in RalA knockdown MEFs (RalAi) expressing HA tagged hRalA* mutant resistant to siRNA (indicated by arrow) (RalAi + hRalA*). Western blot detection of (E) RalA and (F) RalB to confirm the expression of untagged RalA-V23 (WB: RalA) and FLAG-RalBV23 (WB: RalB)(indicated by arrow) respectively. (G, H) Western blot detection of active Ral pulled down with GST-Sec5 (Sec5 PD) from whole cell lysate (WCL) of cells expressing (G) untagged active RalA V23 (WB: RalA), (H) FLAG tagged wild type RalB (RalB WT) and active RalBV23 (RalB V23) (WB: Flag) confirms their activation status. (I) Stable expression of pSuper-shArf6-GFP-Neo plasmid in wild type MEFs detected by GFP fluorescence (GFP) is seen in all cells detected by phase contrast imaging (Phase). Western blot detection of Arf6 (WB: Arf6), RalA (WB: RalA), Caveolin-1 (WB: Cav-1) and β-tubulin (WB: tubulin) in control (CON) and shArf6 knockdown MEFs (shArf6) confirms Arf6 knockdown without affecting other proteins. (J) Cell surface GM1 bound CTxB-Alexa 594 was imaged from suspended control (WTMEF) and GFP shArf6 expressing MEFs (shArf6 MEF) (left panel). Intensity of labeling was quantitated by measuring integrated density for a minimum of 50 cells, calculating their mean and normalizing the same in shArf6 MEF to their respective control (WTMEF). Graph represents mean ± standard error from 3 experiments. (K) FLAG immunoprecipitate from empty FLAG, FLAG-WT and FLAG-V23 RalA (top panel) and RalB (lower panel) expressing HEK293T cells with HA-T157Arf6 were western blotted for FLAG (WB: FLAG-HRP) and Arf6 (WB: HA). Blots are representative of three independent experiments. (L) Representative confocal images showing the colocalization at membrane ruffles in readherent MEFs of CFP tagged WT RalA (CFP-RalA WT) and V23 RalA (CFP-Ra [file mmc1.pdf]
