# Supplementary material for: Ral-Arf6 crosstalk regulates Ral dependent exocyst trafficking and anchorage independent growth signalling
Source: Cell Signal. 2016 Sep;28(9):1225–36. doi: 10.1016/j.cellsig.2016.05.023 (PMC4973806; doi:10.1016/j.cellsig.2016.05.023)
Supplement: Supplementary file 2 — Supplementary sequence information. [file mmc2.pdf]

siRNA sequences used for knockdown.

mouseRalA : 5'-AAGGCAGGTTTCTGTAGAA-3' [1,2],

mouseRalB: 5'-GGTGGTTCTCGACGGAGAA-3' [2,3],

mouseRalBP1: 5'-ACATCATGGTCCTCTCTAC-3' [4],

sh-mouseArf6:5'-AGCTGCACCGCATTATCAA-3' [5,6],

humanRalA: 5'-CAGAGCTGAGCAGTGGAAT-3' [7],

humanRalB: 5'-GGTGATCATGGTTGGCAGC-3' [7],

humanRalBP1: 5'-GTAGAGAGGACCATGATG-3' [8],

human RalBP1#2 : 5'-ACATCATGGTCCTCTCTAC-3' [4],

humanArf6#1: 5'-TCCTCATCTTCGCCAACAA-3' [9] and

humanArf6#2: 5'-GCACCGCATTATCAATGACCG-3' [9]

humanCyth3#1: GTCGCCCAGTTCCTTTATA [10]

humanCyth3#2: CAGCAGAGATCCCTTCTAT [10]

humanCYTH1: AACGACCTCCTGAAGAACACT [11]

humanARNO#2 (Cyth2) : AAGATGGCAATGGGCAGGAAG [11]

siRNA SMARTPOOL SEQUENCES

mouse Sec5 (L-042601)

mSec5_SM1	AGAAGTATTAGGTCGGAAA
mSec5_SM2	TCAACGTACTTCAGCGATT
mSec5_SM3	CAGCAGAGATTACACGTCA
mSec5_SM4	GTGAGTGGCTTGCGCAGTA

Mouse ARNO ((L-059077)

mARNO_SM1	TAAGTGAAGCTATGAGCGA
mARNO_SM2	CAGAAAATTGATCGAATGA
mARNO_SM3	AATTAAGATTCCAGAACGT
mARNO_SM4	GCAAGAAAGAAGCGAATTT

Human ARNO #1 (L-011925)

hARNO_SM1	TGGCAGTGCTCCATGCTTT
hARNO_SM2	AAACCGAACTGCTTTGAAC
hARNO_SM3	GTAAGACCTTGCAACGGAA
hARNO_SM4	GAACACACCCGAGGAGATC

RTPCR primers

mARNO_F: 5'-CCTTTGCCCAGAGATACTGC-3'

mARNO_R: 5'-GGATTGTGAAGGCTGGTGTT-3'

hCYTH1_F: 5'-GTTTGCCCAGCGATATTGTCAG-3'

hCYTH1_R: 5'-GTGCAGACTGGTGTTCAACATG-3'

hARNO_F: 5'-TGGTGGAGAATGAACTGCTG-3'

hARNO_R: 5'-TCATGCAGATCCACAAAAGC-3'

hCYTH3_F: 5'-TGCTACAGAGTTCCCCAGAA-3'

hCYTH3_R: 5'-TTCATCCCTTTCACCCAGGTAG-3'

mActin_F: 5'-GCTACAGCTTCACCACCACA-3'

mActin_R: 5'-TCTCCAGGGAGGAAGAGGAT-3'

hActin_F: 5'-GATTCCTATGTGGGCGAC-3'

hActin_R: 5'-GGTAGTCAGTCAGGTCCCG-3'

Site Directed mutagenesis primers

R79L_A: 5'-GGCAGGAGGACTATGCTGCAATTCTAGACAACTACTTCCGA-3'

R79L_B: 5'-TCGGAAGTAGTTGTCTAGAATTGCAGCATAGTCCTCCTGCC-3'

RalBP1*A: 5'-TTGGAATTCCTTTGGCTGATGCAGTCGAACGAACTATGATGTATGATGGCATTCGGCTGC -3'

RalBP1*B: 5'-GCAGCCGAATGCCATCATACATCATAGTTCGTTCGACTGCATCAGCCAAAGGAATTCCAA -3'

REFERENCES

[1] N. Vitale, J. Mawet, J. Camonis, R. Regazzi, M.-F. Bader, S. Chasserot-Golaz, The Small GTPase RalA controls exocytosis of large dense core secretory granules by interacting with ARF6-dependent phospholipase D1, J. Biol. Chem. 280 (2005) 29921–29928. doi:10.1074/jbc.M413748200.

[2] N. Balasubramanian, J.A. Meier, D.W. Scott, A. Norambuena, M.A. White, M.A. Schwartz, RalA-exocyst complex regulates integrin-dependent membrane raft exocytosis and growth signaling, Curr. Biol. 20 (2010) 75–79. doi:10.1016/j.cub.2009.11.016.

[3] G. Li, L. Han, T.-C. Chou, Y. Fujita, L. Arunachalam, A. Xu, et al., RalA and RalB function as the critical GTP sensors for GTP-dependent exocytosis, Journal of Neuroscience. 27 (2007) 190–202. doi:10.1523/JNEUROSCI.2537-06.2007.

[4] L.E. Goldfinger, C. Ptak, E.D. Jeffery, J. Shabanowitz, D.F. Hunt, M.H. Ginsberg, RLIP76 (RalBP1) is an R-Ras effector that mediates adhesion-dependent Rac activation and cell migration, J. Cell Biol. 174 (2006) 877–888. doi:10.1083/jcb.200603111.

[5] S. Choi, J. Ko, J.-R. Lee, H.W. Lee, K. Kim, H.S. Chung, et al., ARF6 and EFA6A regulate the development and maintenance of dendritic spines, Journal of Neuroscience. 26 (2006) 4811–4819. doi:10.1523/JNEUROSCI.4182-05.2006.

[6] N. Balasubramanian, D.W. Scott, J.E. Casanova, M.A. Schwartz, Arf6 and microtubules in adhesion-dependent trafficking of lipid rafts, Nat. Cell Biol. 9 (2007) 1381–1391. doi:10.1038/ncb1657.

[7] G. Oxford, C.R. Owens, B.J. Titus, T.L. Foreman, M.C. Herlevsen, S.C. Smith, et al., RalA and RalB: antagonistic relatives in cancer cell migration, Cancer Res. 65 (2005) 7111–7120. doi:10.1158/0008-5472.CAN-04-1957.

[8] K.-H. Lim, D.C. Brady, D.F. Kashatus, B.B. Ancrile, C.J. Der, A.D. Cox, et al., Aurora-A phosphorylates, activates, and relocalizes the small GTPase RalA, Mol. Cell. Biol. 30 (2010) 508–523. doi:10.1128/MCB.00916-08.

[9] A. Béglé, P. Tryoen-Tóth, J. de Barry, M.-F. Bader, N. Vitale, ARF6 regulates the synthesis of fusogenic lipids for calcium-regulated exocytosis in neuroendocrine cells, J. Biol. Chem. 284 (2009) 4836–4845. doi:10.1074/jbc.M806894200.

[10] Y. Fu, J. Li, M.-X. Feng, X.-M. Yang, Y.-H. Wang, Y.-L. Zhang, et al., Cytohesin-3 is upregulated in hepatocellular carcinoma and contributes to tumor growth and vascular invasion, Int J Clin Exp Pathol. 7 (2014) 2123–2132.

[11] M. Morishige, S. Hashimoto, E. Ogawa, Y. Toda, H. Kotani, M. Hirose, et al., GEP100 links epidermal growth factor receptor signalling to Arf6 activation to induce breast cancer invasion, Nat. Cell Biol. 10 (2008) 85–92. doi:10.1038/ncb1672.
